# A systematic literature review to evaluate extended dosing intervals in the pharmacological management of acromegaly

**DOI:** 10.1007/s11102-022-01285-1

**Published:** 2022-11-29

**Authors:** M. Fleseriu, Z. Zhang, K. Hanman, K. Haria, A. Houchard, S. Khawaja, A. Ribeiro-Oliveira, M. Gadelha

**Affiliations:** 1 Pituitary Center at Oregon Health & Science University, Portland, OR USA; 2 Department of Endocrinology and Metabolism, Huashan Hospital, Shanghai Medical College, Fudan University, Shanghai, China; 3 Costello Medical, London, UK; 4 Ipsen Pharma, Boulogne-Billancourt, France; 5 World Alliance of Pituitary Organizations, Zeeland, The Netherlands; 6 Ipsen, Cambridge, MA USA; 7 Neuroendocrinology Research Center/Endocrinology Division, Medical School and Hospital Universitário Clementino Fraga Filho, Universidade Federal do Rio de Janeiro, Rio de Janeiro, Brazil

**Keywords:** Acromegaly, Extended dosing intervals, Growth hormone, Somatostatin receptor ligand, Pituitary adenoma

## Abstract

**Purpose:**

This systematic literature review investigated whether extended dosing intervals (EDIs) of pharmacological acromegaly treatments reduce patient burden and costs compared with standard dosing, while maintaining effectiveness.

**Methods:**

MEDLINE/Embase/the Cochrane Library (2001–June 2021) and key congresses (2018–2021) were searched and identified systematic literature review bibliographies reviewed. Included publications reported on efficacy/effectiveness, safety and tolerability, health-related quality of life (HRQoL), and patient-reported and economic outcomes in longitudinal/cross-sectional studies in adults with acromegaly. Interventions included EDIs of pegvisomant, cabergoline, and somatostatin receptor ligands (SRLs): lanreotide autogel/depot (LAN), octreotide long-acting release (OCT), pasireotide long-acting release (PAS), and oral octreotide; no comparator was required.

**Results:**

In total, 35 publications reported on 27 studies: 3 pegvisomant monotherapy, 11 pegvisomant combination therapy with SRLs, 9 LAN, and 4 OCT; no studies reported on cabergoline, PAS, or oral octreotide at EDIs. Maintenance of normal insulin-like growth factor I (IGF-I) was observed in ≥ 70% of patients with LAN (1 study), OCT (1 study), and pegvisomant monotherapy (1 study). Achievement of normal IGF-I was observed in ≥ 70% of patients with LAN (3 studies) and pegvisomant in combination with SRLs (4 studies). Safety profiles were similar across EDI and standard regimens. Patients preferred and were satisfied with EDIs. HRQoL was maintained and cost savings were provided with EDIs versus standard regimens.

**Conclusions:**

Clinical efficacy/effectiveness, safety, and HRQoL outcomes in adults with acromegaly were similar and costs lower with EDIs versus standard regimens. Physicians may consider acromegaly treatment at EDIs, especially for patients with good disease control.

**Supplementary Information:**

The online version contains supplementary material available at 10.1007/s11102-022-01285-1.

## Plain language summary

Acromegaly is a rare disease where the body makes too much growth hormone. It is usually caused by a benign pituitary tumor. Excess growth hormone causes body parts such as the hands, feet, head, and heart to grow larger than normal. Organs including the heart can work less well, leading to other health conditions. Treatment aims to improve signs and symptoms, decrease tumor size, and normalize growth hormone levels. However, if surgery does not cure acromegaly, it can require ongoing, life-long treatment. Treatment can cause frequent side effects and impact daily life. Increasing time between medication doses could ease the burden of acromegaly treatment and reduce costs.

We searched scientific literature for evidence on less frequent dosing (known as extended dosing intervals) when treating acromegaly, investigating efficacy/effectiveness, safety, well-being, and costs. We searched for evidence on treatments including: lanreotide autogel/depot, octreotide long-acting release, oral octreotide, pasireotide long-acting release, cabergoline, and pegvisomant.

In people with stable acromegaly at standard dosing of lanreotide autogel, octreotide long-acting release, and pegvisomant, less frequent dosing generally did not compromise treatment effectiveness and kept growth hormones at normal levels. Furthermore, monthly treatments with lanreotide autogel or octreotide long-acting release alone are sometimes not enough to normalize hormone levels. In these cases, adding pegvisomant with less frequent dosing generally helped to stabilize disease.

Generally, patient quality of life did not deteriorate with less frequent dosing, and satisfaction remained high. Furthermore, patients preferred less frequent dosing compared to standard dosing, which was also shown to reduce treatment costs.

## Introduction

Acromegaly is a rare disorder characterized by excessive growth of bodily tissues, usually caused by a benign pituitary adenoma [1]. Typical symptoms of acromegaly include the coarsening of facial features and the enlargement of hands, feet, and internal organs, which can lead to other comorbidities [1–4]. Treatment is aimed at normalizing growth hormone (GH) and insulin-like growth factor I (IGF-I) levels, controlling tumor mass, improving associated signs and symptoms, and preventing complications such as cardiovascular disease and respiratory disease [5–8]. Pharmacological treatment is divided into three principal classes: somatostatin receptor ligands (SRLs), GH-receptor antagonists, and dopamine agonists [9, 10].

SRLs such as injectable lanreotide autogel/depot (LAN), octreotide long-acting release (OCT), and pasireotide long-acting release (PAS) have a recommended dosing interval of every 4 weeks [11–14], while oral octreotide is typically administered twice daily [15]. These therapies block GH secretion by binding to somatostatin receptors, mimicking endogenous somatostatin [9, 16, 17].

Pegvisomant is an injectable GH-receptor antagonist with a recommended daily dosing regimen [18], and is commonly administered in off-label combinations with other classes of pharmacological therapy [17, 19]. Cabergoline is a dopamine agonist which inhibits GH secretion in acromegaly, and is prescribed as off-label in the United States (US) up to once daily, sometimes in combination with SRLs and pegvisomant [20].

Given the chronic nature of acromegaly and the potential requirement for life-long therapy, reducing the frequency of dosing may lessen the burden of treatment for patients. LAN has already been approved for administration at extended dosing intervals (EDIs) of 6 and 8 weeks in Europe, the US, and some countries in Asia [11, 12, 21], while other treatments such as OCT are still only licensed at standard intervals. However, the implications of EDIs for the maintenance of clinical efficacy (and effectiveness, in the “real-world” setting [22]) tolerability, and health-related quality of life (HRQoL) have not yet been fully elucidated. The objective of this systematic literature review was therefore to evaluate clinical efficacy/effectiveness, safety, and tolerability, HRQoL, treatment preference, and economic outcomes in patients with acromegaly receiving treatment at EDIs for the three principal classes of pharmacological therapies.

## Methods

A pre-specified protocol was followed in this systematic literature review, details of which were registered on PROSPERO 2021: CRD42021278922 [23]. The systematic literature review was conducted and reported in line with the Preferred Reporting Items for Systematic Review and Meta-Analysis (PRISMA) guidelines [24].

### Search strategy

The MEDLINE, Ovid MEDLINE® and Epub Ahead of Print, In-Process, In-Data-Review & Other Non-Indexed Citations and Daily, Embase (all searched via Ovid SP), and the Cochrane Library [including Cochrane Database of Systematic Reviews (CDSR) and Cochrane Central Register of Controlled Trials (CENTRAL), searched via Wiley Online] electronic databases were searched on June 30, 2021. The databases searched were those recommended in the Cochrane Handbook for Systematic Reviews of Interventions [25], and were expected to capture articles including those indexed in the Web of Science, Cumulative Index to Nursing and Allied Health Literature (CINAHL), and EBSCOhost databases. Searches were conducted systematically using terms grouped as acromegaly-related, treatment-related, or exclusion search terms (full list of search terms presented in Supplementary Table 1–Supplementary Table 3). Since guidelines for managing acromegaly have been updated over time [5, 6, 17], the databases were searched for articles published since 2001, to ensure only the most relevant and recent data were captured. The majority of treatments studied in this review were approved in the US and Europe after 2001. Therefore, it is unlikely that relevant data will have been published prior to 2001, and articles published prior to 2001 would have been less relevant to this review.

Relevant congresses which had taken place in 2018–2021 were hand-searched for further evidence (Supplementary Table 4). Congresses were selected for quality, coverage of key regions, and availability of abstracts in English. The bibliographies of relevant systematic literature reviews and meta-analyses identified during electronic database and congress searching were also hand-searched to identify additional relevant studies for inclusion; these systematic literature reviews and meta-analyses were subsequently excluded. As it was expected that there was a minimal risk of missing relevant articles following searches of the online databases, congresses, and reference lists, manual searches of Google Scholar were not conducted.

### Study selection, data extraction, and quality assessment

The Cochrane Collaboration recommendations for stringent article screening were followed [26]. Studies eligible for inclusion in this systematic literature review assessed adult patients with a confirmed diagnosis of acromegaly treated with EDIs of LAN, OCT, oral octreotide, PAS, cabergoline, and pegvisomant. Standard dosing regimens in this systematic literature review were considered to be administration once every 4 weeks for LAN, OCT, and PAS, twice daily for oral octreotide, twice weekly for cabergoline, and once daily for pegvisomant [11–15, 18, 27–31]. EDIs were therefore defined, for the purposes of this systematic literature review, as administration less often than every 4 weeks for LAN, OCT, and PAS, less often than twice daily for oral octreotide, less often than twice weekly for cabergoline, and less often than once daily for pegvisomant. Eligible studies reported on relevant clinical efficacy/effectiveness, safety and tolerability (including adherence), HRQoL, treatment preference, satisfaction, and economic and healthcare resource use outcomes. Interventional and observational (cohort, case-control, and cross-sectional) studies were considered for inclusion with no comparator treatment required, and review articles were excluded. Full eligibility criteria are detailed in Supplementary Table 5.

Two independent reviewers screened titles and abstracts against the pre-specified eligibility criteria with discrepancies resolved either by reaching consensus, or by the decision of a third reviewer. For any titles and abstracts that were determined to be potentially relevant to the systematic literature review, this process was then repeated using the full texts. Articles reporting on the same study were considered as one unique study for extraction and synthesis, with the article reporting the main results of the study considered as the primary article, and additional articles considered as secondary articles.

Data extraction of study characteristics, patient characteristics, and results was performed in line with guidelines from the University of York Centre for Reviews and Dissemination (CRD [32]; full list of extracted data in Supplementary Table 6). Data extraction into a pre-specified extraction grid and quality assessment were performed by a single reviewer and independently verified by a second reviewer. The quality of primary studies was assessed with the Alberta Heritage Foundation for Medical Research (AHFMR) checklist for quantitative studies [33], a single tool which incorporates study design into determining study quality, and hence allows objective comparison of risk of bias across studies on a single scale.

Economic costs extracted from the studies were converted to US dollars (USD), utilizing the XE.com historical rate tables [34]. Costs were converted using exchange rates from January 1st of the year that data collection took place (or the publication year if the year of data collection was not reported).

### Feasibility assessment for meta-analysis

A feasibility assessment was conducted to assess the suitability of combining estimates reported in the included studies for each outcome into pooled estimates in a meta-analysis. The following efficacy/effectiveness outcomes of interest were assessed for each intervention in turn: IGF-I levels, GH levels, biochemical control, IGF-I control, and tumor size. For each outcome, heterogeneity in outcome definition and comparability of units of reporting, biochemical assays used, treatment dosing and frequency, sample sizes, and timepoints reported were investigated across studies.

## Results

### Characteristics of included studies

**Fig. 1:**
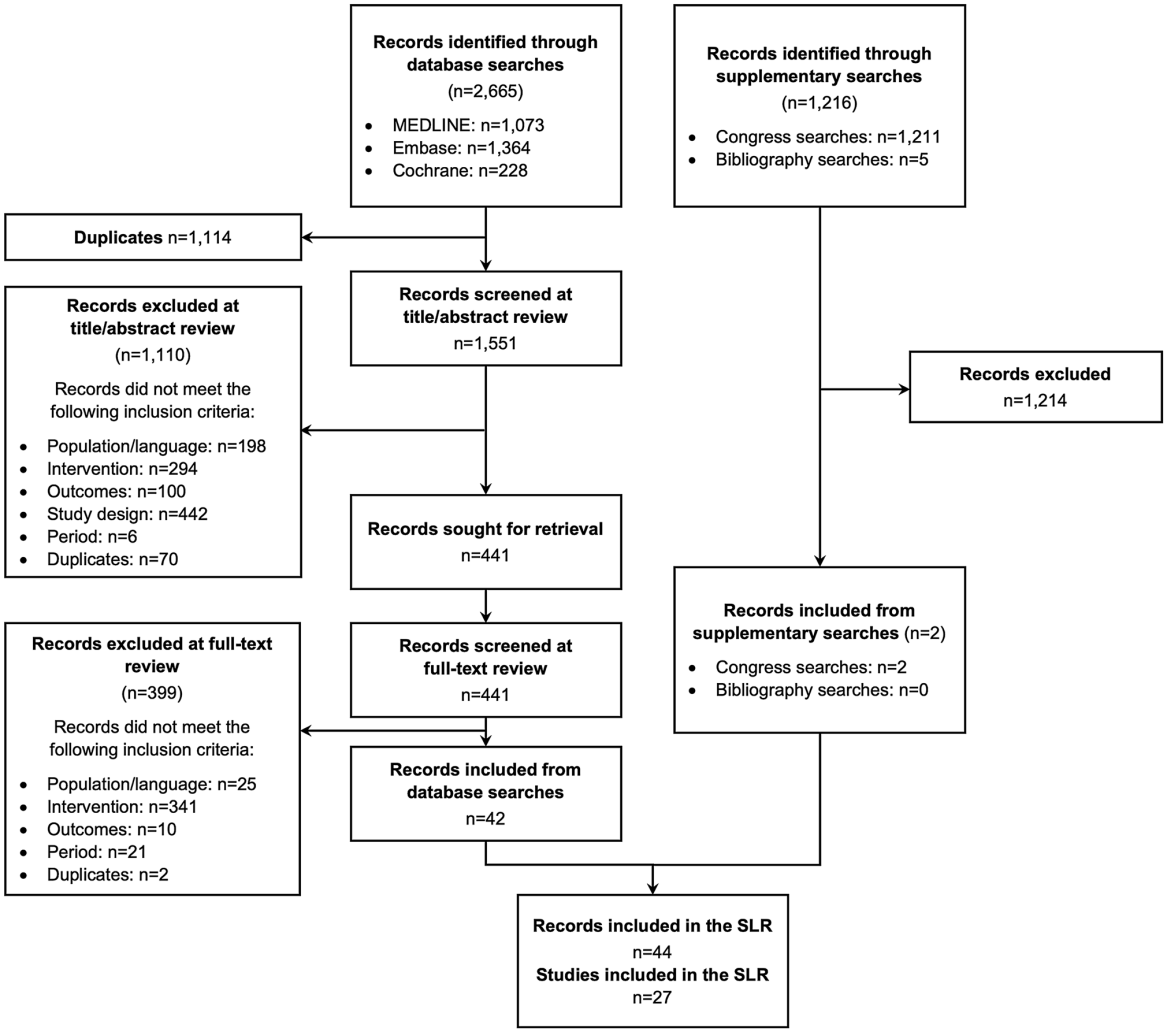
PRISMA flowchart. Reasons for exclusion of records included: population (results not reported for adults with acromegaly), language (article not in English), intervention [intervention or dosing interval reported is not included in the scope of the review (e.g., only standard intervals assessed, or treatment not listed in Supplementary Table 5)], outcomes [no outcomes of interest reported (e.g., genetic studies)], and period [paper published outside of the date limit of interest (i.e., before 2001)]. *MEDLINE* medical literature analysis and retrieval system online, *PRISMA* preferred reporting items for systematic reviews and meta-analyses

A total of 44 relevant articles were included from the electronic database and hand searches (Fig. 1). Nine articles that either included ≤ 5 patients treated with EDIs, were treatment withdrawal studies, or reported relevant information only as part of patients’ baseline characteristics were deprioritized for extraction to ensure that the most robust and relevant evidence was captured (Supplementary Table 7). The remaining 35 articles, comprising 27 unique primary studies and 8 secondary articles, were ultimately included.

**Fig. 2:**
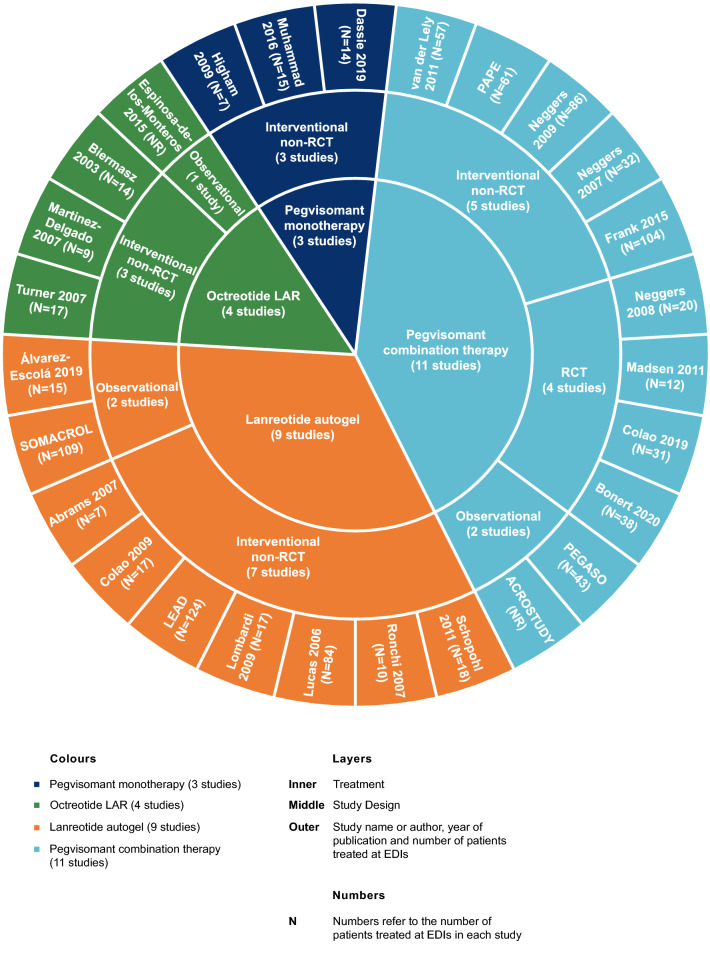
Summary of study characteristics. Studies are shown with the name of the first author and year, except where the study was given a name (e.g., LEAD). “Pegvisomant combination therapy” refers to pegvisomant in combination with either lanreotide autogel or octreotide LAR. *EDI* extended dosing interval, *LAR* long-acting release, *NR* not reported, *RCT* randomized controlled trial

Pegvisomant as a monotherapy (n = 3 studies [35–37]), pegvisomant in combination with standard dosing intervals of LAN or OCT (n = 11, henceforth termed as “pegvisomant combination therapy” [38–48]), LAN (n = 9 [49–57]), and OCT (n = 4 [58–61]) were investigated at EDIs in the included studies (Fig. 2). No studies reporting on oral octreotide, cabergoline, or PAS administered at EDIs were identified. The majority of studies were interventional (n = 21 [35, 36, 38, 40, 41, 43–49, 52–58, 60, 61]), of which 16 were interventional non-randomized controlled trials. Characteristics of each study are presented in Supplementary Table 8.

### Baseline characteristics of patients treated at EDIs

Where studies reported baseline characteristics for both EDI and non-EDI treatment arms, these have been presented in Supplementary Table 9. Across all studies, 7–109 patients were treated at EDIs and 13/27 studies featured < 30 patients treated at EDIs [35–37, 43, 46, 49, 50, 52, 56–58, 60, 61], while 3/26 included 6–10 patients treated at EDIs [36, 49, 60]. One study reported that > 5 patients were treated with EDIs, but did not report the precise number [42]. At baseline, a mixture of patients with biochemically controlled and uncontrolled disease was observed between and within studies. Though not frequently reported, median time since diagnosis of acromegaly varied from 1.4 (interquartile range 0.9–3.5 years) to approximately 14 years (range 1.5–27.1 years) across studies in which patients were not newly diagnosed. The majority of studies reported that patients had previously received pharmacological treatment for acromegaly at standard or unreported intervals: SRLs (n = 22 [36, 38–51, 55–61]), pegvisomant monotherapy (n = 4 [36, 38, 48, 51]), and pegvisomant combination therapy (n = 2 [37, 51]). Median duration of previous treatment was not systematically reported, but studies often required that patients were receiving their previous treatment for ≥ 6 months to be eligible for study entry. Across studies that included patients with biochemical control (i.e., control of IGF-I and/or GH levels, as was defined by each study) at baseline, patients were usually required to have maintained biochemical control for ≥ 6 months prior to study initiation in order to be eligible to enter each study. However, duration of prior control was infrequently reported across studies.

### SRLs

#### IGF-I and GH

**Fig. 3:**
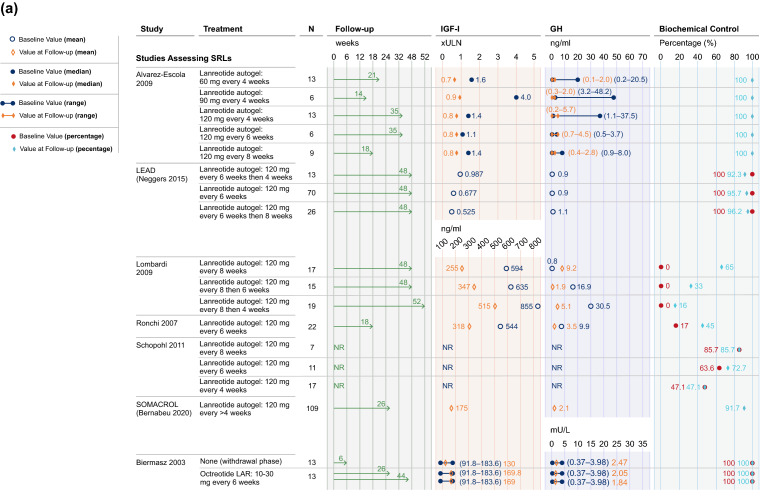

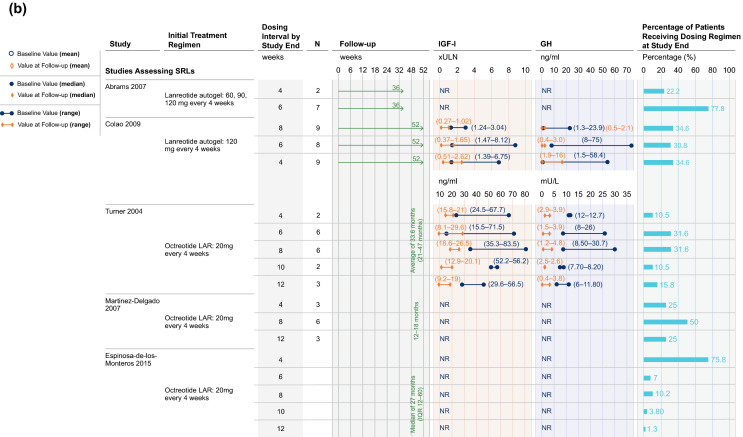

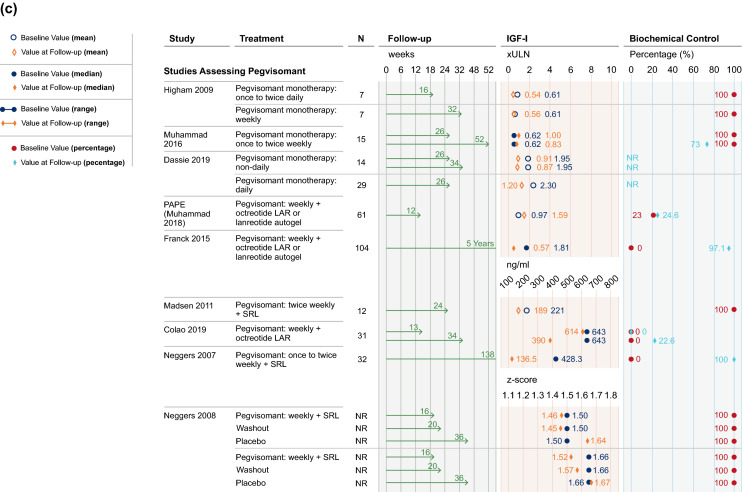
**a** IGF-I and GH results, studies assessing SRLs. In Alvarez-Escola 2019, patients were retrospectively selected based on achievement of biochemical control. Controlled IGF-I and GH typically referred to IGF-I levels being within (or close to) age- and sex-adjusted reference ranges [e.g., < 1.0 or 1.2 × upper limit of normal (ULN)], and GH below a threshold value (e.g., ≤ 2.5 μg/L). Data have been presented for Period 1 of the LEAD study (Neggers 2015). Biochemical control has been presented as IGF-I control where possible; if data on control of IGF-I levels were not available, biochemical control is displayed as GH control or control of GH and IGF-I combined. In studies where individual patient data has been reported, IGF-I and GH levels have been provided as a range and a decrease has been interpreted if the entire range at follow-up has shifted to cover lower hormonal levels compared to baseline. **b** IGF-I and GH results, systematic dose extension studies assessing SRLs. Controlled IGF-I and GH typically referred to IGF-I levels being within (or close to) age- and sex-adjusted reference ranges [e.g., < 1.0 or 1.2 × upper limit of normal (ULN)], and GH below a threshold value (e.g., ≤ 2.5 μg/L). **c** IGF-I results, studies assessing pegvisomant. Controlled IGF-I and GH typically referred to IGF-I levels being within (or close to) age- and sex-adjusted reference ranges [e.g., < 1.0 or 1.2 × upper limit of normal (ULN)], and GH below a threshold value (e.g., ≤ 2.5 μg/L). For PAPE (Muhammad 2018), the percentage of patients with biochemical control was calculated from reported data (14/61). *GH* growth hormone, *IGF-I* insulin-like growth factor I, *IQR* interquartile range, *LAR* long-acting release, *NR* not reported, *SRL* somatostatin receptor ligand, *ULN* upper limit of normal

Studies reporting on IGF-I and GH outcomes in patients treated with SRLs had varied study designs. Results for studies using defined treatment arms with stable dosing intervals until study end are presented in Fig. 3a, while results for studies using systematic dose extension regimens based on achievement of biochemical control are displayed in Fig. 3b.

At study end, IGF-I levels decreased from baseline in all four studies assessing IGF-I following treatment with LAN at EDIs [52, 53, 55, 56]. Across these studies, no patients (n = 2 studies [52, 53]), all patients (n = 1 [55]) and an unreported proportion of patients (n = 1 [56]) had controlled IGF-I and/or GH at baseline. Definitions of controlled IGF-I and GH varied across studies and typically referred to IGF-I levels being within (or close to) age- (and occasionally sex-) adjusted reference ranges [e.g., < 1.0 or 1.2× upper limit of normal (ULN)], and GH below a threshold value (e.g., ≤ 2.0 μg/L, ≤ 2.5 μg/L, or ≤ 5 mU/L). In this systematic literature review, control is reported as was defined by each individual study.

Control of IGF-I levels was reported at study end with LAN administered at EDIs in ≥ 70% of patients in all four studies in which some, all or an unreported proportion of patients had controlled IGF-I at baseline [49, 51, 55, 57]. Additionally, control of IGF-I levels was achieved with LAN at EDIs by study end in 33%–65% of patients in studies where no patients had controlled IGF-I levels at baseline (n = 2/2 studies [52, 53]).

GH levels decreased from baseline to study end with EDIs of LAN in all three studies which reported on GH, regardless of the proportion of patients with controlled IGF-I and/or GH at baseline [52, 53, 56]. These three studies also reported decreased IGF-I with LAN, highlighting the efficacy of EDIs of LAN in decreasing both IGF-I and GH levels. Patients generally maintained or achieved normal GH levels by study end, regardless of baseline biochemical control status (n = 5/7 studies), with no obvious difference between 6- and 8-week dosing intervals [49, 51–53, 55–57].

Controlled IGF-I was maintained in 69–75% of patients treated with OCT at EDIs in studies where all patients had normal IGF-I at baseline (2/2 studies [58, 60]). In a further study where the proportion of patients with IGF-I control was not reported at baseline, 22% of patients had controlled IGF-I at study end [59]. In the final study where no patients had normal IGF-I levels at baseline, a decrease in IGF-I levels was observed, though the resultant proportion of patients with control of IGF-I was not reported [61].

Normalized GH levels at study end were reported following treatment with OCT at EDIs, where 0% (1/1 study) and 100% (2/2 studies) of patients had baseline biochemical control [58, 60, 61]. Decreased GH levels at study end were observed in one study where no patients had baseline control and one of the studies where all patients had controlled disease at baseline [58, 61].

#### Tumor size

Tumor shrinkage was observed at study end in 17/17 treatment-naïve patients whose dose interval of LAN was extended from the initial 4-weekly dosing based on GH normalization (≤ 2.5 μg/L) (Table 1) [52]. The study did not comment on the possible carry-over effect from the initial 4-weekly dosing or the clinical significance of the observed tumor shrinkage in the patients who were treated at EDIs. No studies reporting on OCT assessed tumor size.

**Table 1:** Summ﻿ar﻿y of additional efficacy/effectiveness results

Study	Treatment		N	Definition of GH control	n/N (%) with GH control	Tumor size, n/N (%)	Other information
*Studies assessing SRLs*							
Lombardi 2009 [53]	LAN 120 mg every 8 weeks		17	GH levels ≤ 2.5 μg/L	Baseline: 0/17 (0) Week 48: 17/17 (100)	NR	Dosing interval shortened in patients with high GH levels when treated at 8-week intervals. p < 0.001 for GH normalization
	LAN 120 mg every 8 weeks then 6 weeks		15	GH levels ≤ 2.5 μg/L	Baseline: 0/15 (0) Week 48: 11/15 (73)		
	LAN 120 mg every 8 weeks then 4 weeks		19	GH levels ≤ 2.5 μg/L	Baseline: 0/19 (0) Week 52: 4/19 (21)		
Colao 2009^a^ [52]	LAN 120 mg every 8 weeks		9	All patients had abnormal IGF-I at baseline and were started on 4-weekly dosing of LAN 120 mg. Dosing intervals were extended based on GH levels (≤ 2.5 μg/L). Dosing intervals were able to be extended to 6 or 8 weeks in 17/26 patients at 12 months; 9/26 patients required dosing every 4 weeks		Week 52: tumor volume shrinkage in 9/9 (100)	EDIs (6/8 weeks) assigned to patients with lower GH levels. Ranges are taken from individual patient data
	LAN 120 mg every 6 weeks		8			Week 52: tumor volume shrinkage in 8/8 (100)	
	LAN 120 mg every 4 weeks		9			Week 52: tumor volume shrinkage in 8/9 (NR), no change in 1/9 (NR)	
Alvarez-Escola 2019^b^ [50]	LAN 60 mg every 4 weeks		13	In this retrospective study, patients were chosen based on achievement of GH levels < 2.5 ng/mL and/or normalized IGF-I levels following treatment with LAN. Thus, all patients in this study had biochemical control at study end. All patients had GH levels ≥ 2.5 ng/mL and/or non-normalized IGF-I levels at baseline		NR	NA
	LAN 90 mg every 4 weeks		6				
	LAN 120 mg every 4 weeks		13				
	LAN 120 mg every 6 weeks		6				
	LAN 120 mg every 8 weeks		9				
LEAD (Neggers 2015) [55]	Phase 1	LAN 120 mg every 6 weeks	124	GH ≤ 2.5 μg/L	Week 24 (Phase 1): 105/112 (93.8) [95% CI 89.3–98.2] with data available	NR	124 patients started the 6-weekly dosing phase (Phase 1) and 109 of these patients continued into Phase 2 with the different dosing schedules (15 withdrawn prematurely) Only 112 patients had data available at Week 24
				Normal IGF-I levels and GH ≤ 2.5 μg/L	Week 24 (Phase 1): 103/112 (92.0) [95% CI 86.9–97.0] with data available	NR	
	Phase 2	LAN 120 mg 4 weeks	13	GH ≤ 2.5 μg/L	Week 48: NR (100)	NR	Shorter dosing intervals were given to patients with higher IGF-I. Change from baseline in IGF-I amongst patients with normalized IGF-I: + 0.248 × ULN
		LAN 120 mg every 6 weeks	70	GH ≤ 2.5 μg/L	Week 48: NR (92.6)		Two patients withdrew prematurely from the 6-week treatment arm Shorter dosing intervals were given to patients with higher IGF-I levels (patients with lowest IGF-I were dosed at the longest intervals). Change from baseline in IGF-I amongst patients with normalized IGF-I: + 0.061 × ULN 6- and/or 8-week EDI groups had smaller increases in IGF-I than 4-week intervals: p = 0.0013
		LAN 120 mg every 8 weeks	26	GH ≤ 2.5 μg/L	Week 48: NR (96.2)		Shorter dosing intervals were given to patients with higher IGF-I. Change from baseline in IGF-I amongst patients with normalized IGF-I: − 0.179 × ULN Larger decrease in IGF-I than 4-week intervals: p < 0.0001
Schopohl 2011 [57]	LAN 120 mg every 8 weeks		7	GH < 2.0 ng/mL	Baseline: 7/7 (100) One injection interval after the 6th injection: 7/7 (100)	NR	Patients were assigned to EDIs based on previously receiving a lower octreotide LAR dose, and dosing intervals were then adjusted based on IGF-I level
	LAN 120 mg every 6 weeks		11	GH < 2.0 ng/mL	Baseline: 8/11 (72.7) One injection interval after the 6th injection: 8/11 (72.7)		
	LAN 120 mg every 4 weeks		17	GH < 2.0 ng/mL	Baseline: 13/17 (76.5) One injection interval after the 6th injection: 10/17 (58.8)		
SOMACROL (Bernabeu 2020) [51]	LAN 120 mg every > 4 weeks		109	GH ≤ 2.5 ng/mL	Month 6 after LAN EDI treatment: NR (80.6)	NR	Cross-sectional study. 77.1% of patients were taking concomitant medication. 57.8%, 38.5% and 2.8% of patients had dosing intervals of 5–6 weeks, 7–8 weeks and > 8 weeks, respectively. All patients in the present study presented normal IGF-I levels at initiation of the EDI
Abrams 2007^a^ [49]	All 9 patients had controlled IGF-I at baseline, and 7/9 had GH < 1.7 ng/mL. Dosing intervals of LAN 120 mg were extended beyond 4 weeks (baseline) based on normal IGF-I and GH levels < 1.7 μg/L. Overall, the dosing interval could be extended to 6 weeks in 7/9 patients by Week 36 without deteriorating the biochemical control of acromegaly; the remaining 2/9 patients required 4-weekly dosing						
Biermasz 2003 [58]	Withdrawal phase: no treatment		14	GH < 5 mU/L	Week 6: 13/14	NR	One patient withdrew at Week 26 and continued with a 4-week injection interval No significant increase in IGF-I levels
	OCT 10–20 mg every 6 weeks			GH < 5 mU/L	Week 26: 13/14 Week 44: 13/13 (100)		
Turner 2004^a,c^ [61]	OCT 20–30 mg every 4 weeks		2	Lack of control: GH > 5 mU/L and elevated IGF-I levels	NR	NR	Patients had GH > 5 mU/L and elevated IGF-I levels at baseline and were started on OCT 20 mg every 4 weeks. Dosing intervals were extended based on achievement of GH < 5 mU/L GH and IGF-I were lower on treatment compared with baseline (p < 0.01)
	OCT 20–30 mg every 6 weeks		6				
	OCT 20 mg every 8 weeks		6				
	OCT 20–30 mg every 10 weeks		2				
	OCT 20–30 mg every 12 weeks		3				
Martinez-Delgado 2007^a^ [60]	Patients had controlled IGF-I and GH at baseline, and were started on OCT 20 mg every 4 weeks. Dosing intervals were extended in patients with safe GH (< 2.5 ng/mL) and IGF-I levels. By study end (12–18 months), EDIs (6–8 weeks) were employed in 9/12 (75%) of patients; 3/12 (25%) patients required 4-weekly dosing						
Espinosa-de-los-Monteros 2015^a^ [59]	Patients were started on OCT 20 mg every 4 weeks (proportion of patients with biochemical control at baseline not reported). Dosing intervals were extended in patients according to treatment response (GH < 2.5 ng/mL and IGF-I < 1.2 × ULN). After ≥ 3 months, EDIs (6–12 weeks) were employed in 22.3% of patients						
*Studies assessing pegvisomant*							
Higham 2009 [36]	Pegvisomant monotherapy 10–20 mg twice weekly		7	NR	NR	NR	Patients were previously on daily pegvisomant
	Pegvisomant monotherapy 10–20 mg weekly						
Muhammad 2016 [37]	Pegvisomant monotherapy 60 mg (starting dose) once or twice weekly		15	NR	NR	Month 12: no change in tumor size in 15/15 (100)	Patients were previously treated with SRLs to achieve disease control
Dassie 2019 [35]	Pegvisomant monotherapy 12 mg non-daily		14	NR	NR	NR	Patients were previously on daily pegvisomant. 7–14% of patients were on combination therapy
	Pegvisomant monotherapy 24 mg daily		29				NA
Madsen 2011 [43]	Pegvisomant 15–30 mg twice weekly + OCT or LAN at half usual dosage every 4 weeks		12	NR	NR	NR	NA
PAPE (Muhammad 2018) [44]	Pegvisomant 61–134 mg weekly + LAN 120 mg or OCT 30 mg every 4 weeks		61	GH ≤ 2.5 μg/L	Baseline: 10/61 (NR) Week 12: 11/61 (NR)	NR	Patients previously had well controlled acromegaly with SRLs; pegvisomant dose was halved over 12 weeks
Colao 2019 [40]	Baseline to Month 3: OCT 40 mg every 4 weeks Months 3–8: Pegvisomant 70 mg weekly + OCT 40 mg every 4 weeks		31	GH ≤ 2.5 and IGF-I within normal range adjusted for age and gender	Month 3: 0/31 (0)	NR	Patients’ disease was previously inadequately controlled with SRL treatment (non-EDI)
				GH > 2.5 μg/L to < 5 μg/L and either ≥ 50% decrease in IGF-I compared with baseline or IGF-I within the normal range, or mean GH < 2.5 μg/L and ≥ 50% decrease in IGF-I compared with baseline and IGF-I > 1 × ULN	Month 8: 7/31 (22.6) [95% CI 9.6–41.1]		
Franck 2015 [41]	Pegvisomant 80 mg weekly + OCT 30 mg or LAN 120 mg every 4 weeks		104	NR	NR	Median follow-up of 5 years: > 20% tumor volume shrinkage in 13/104 (12.5) Tumor growth in 1/104 (1)	Patients previously had uncontrolled disease with SRLs (at standard intervals)
Neggers 2007 [47]	Pegvisomant 60 mg once or twice weekly (40 mg starting dose) +﻿ LAN 120 mg or OCT 30 mg every 4 weeks		32	NR	NR	Median follow-up of 2.7 years: Tumor volume shrinkage > 25% in 4/32 (13) No tumor volume growth in 28/32	Patients previously had uncontrolled disease with SRLs No patients with tumor shrinkage had previously received radiotherapy, and one underwent transsphenoidal surgery
Neggers 2009^d^ [45]	Pegvisomant 20–200 mg weekly or twice weekly + OCT or LAN (dose not reported)		86	NR	NR	Mean follow-up of 2.4 years: Tumor volume shrinkage > 20% in 14/74 (19) Tumor volume increase in 0/74 (0)	Patients were previously on SRL therapy (n = 63 did not have disease control) Tumor size could not be assessed in 12/86 patients Tumor volume shrinkage by > 20% was considered to be clinically significant. Two patients with tumor shrinkage previously underwent transsphenoidal surgery and one had also received radiotherapy. However, patients with the highest percentage of tumor shrinkage were receiving primary medical treatment (definition not provided by study). The observation of tumor shrinkage was therefore noted to be most likely the effect of continuous treatment with primary, long-acting SRL
Bonert 2020 [38]	Pegvisomant weekly 40–160 mg + LAN 120 mg or OCT 30 mg every 4 weeks		15	NR	NR	NR	p > 0.99 for all comparisons of proportions of patients with IGF-I control by study end between treatment arms
	Pegvisomant weekly 40–160 mg + LAN 60 mg or OCT 10 mg every 4 weeks		23				
	Pegvisomant daily 15–60 mg + LAN 60 mg or OCT 10 mg every 4 weeks		14				
van der Lely 2011 [48]	Overall population: Pegvisomant 40–80 mg weekly to twice weekly + LAN 120 mg every 4 weeks		57	NR	NR	Week 28: Tumor size shrinkage > 20% in 7/53 (13.2) Tumor size growth > 20% in 13/53 (24.5)	Study reports it was not designed to examine changes in tumor size. Most patients had microadenomas or had undergone tumor debulking surgery. Study reports that SRL-induced tumor shrinkage was likely to be maximal during prior SRL treatment rather than with pegvisomant combination therapy at EDIs
	Pegvisomant 40 mg weekly + LAN 120 mg every 4 weeks		13				
	Pegvisomant 60 mg weekly + LAN 120 mg every 4 weeks		13				
	Pegvisomant 80 mg weekly + LAN 120 mg every 4 weeks		16				
	Pegvisomant 40 mg twice weekly + LAN 120 mg every 4 weeks		5				
	Pegvisomant 60 mg twice weekly + LAN 120 mg every 4 weeks		10				

*CI* confidence interval, *EDI* extended-dosing interval, *GH* growth hormone, *IGF-I* insulin-like growth factor I, *LAN* lanreotide autogel/depot, *LAR* long-acting release, *OCT* octreotide long-acting release, *NA* not applicable, *NR* not reported, *SRL* somatostatin receptor ligand

^a^In these studies, patients all started on the same dosing regimen, and then dosing intervals were systematically altered based on IGF-I and/or GH levels (e.g., intervals were extended if biochemical control was achieved/maintained). Therefore, EDIs were not attempted in all patients, so the level of control with EDIs cannot be concluded

^b^Patients were selected for this retrospective study based on achievement of biochemical control. Therefore, this study exhibits selection bias as 100% of patients will have had biochemical control by study end

^c^Individual patient level data are reported by the study and are presented here as ranges

^d^Dosing regimen of SRL therapy is not reported

#### Safety and tolerability outcomes

Five interventional studies reported on adverse events (AEs) during treatment with SRLs at EDIs [40, 43, 53, 55, 57]. An AE was reported by 70.0–87.5% of patients treated with SRLs at EDIs, with little variation observed between extended and standard dosing intervals (n = 2 studies reporting the total proportion of patients with AEs of any severity and organ class [55, 57]; Table 2). In general, AEs were mild to moderate [40, 55, 57]. Serious AEs and serious treatment-related AEs (TRAEs) were experienced by a small proportion of patients across all treatment regimens (n = 3 studies [40, 53, 55]). The most commonly observed gastrointestinal event was diarrhea [55, 57], with no clear trend in incidence observed between EDI and standard dosing regimens (n = 2 studies [55, 57]). No study reported deaths that were related to treatment at either standard or EDI regimens (n = 3 studies).

**Table 2:** Summary of safety results

Study	Treatment	N	n/N (%) patients experiencing any AE	n/N (%) patients experiencing TRAE	n/N (%) patients experiencing serious AE	n/N (%) patients adhering to treatment	Other information	Study type
*Studies assessing SRLs*								
Alvarez-Escola 2019 [50]	LAN 60 mg every 4 weeks	13	NR	NR	NR	Mean follow-up 5.7 years: NR (92.3)	Adherence was determined by the number of omitted doses and by patients’ continuation of the treatment at the end of the study, collected from clinical history	Observational
	LAN 90 mg every 4 weeks	6				Mean follow-up 5.7 years: NR (83.3)		
	LAN 120 mg every 4 weeks	13				Mean follow-up 5.7 years: NR (84.6)		
	LAN 120 mg every 6 weeks	6				Mean follow-up 5.7 years: NR (66.7)		
	LAN 120 mg every 8 weeks	9				Mean follow-up 5.7 years: NR (77.8)		
SOMACROL (Bernabeu 2020) [51]	LAN 120 mg every > 4 weeks	109	NR	NR	NR	Month 6: 103/109 (94.5)	Non-adherence was measured as ≥ 1 missed injection during the study period observed from clinical records. No statistically significant differences were observed in adherence rates between the patient group treated every 5–6 weeks versus those treated every 7–8 weeks	Observational
LEAD (Neggers 2015) [55]	LAN 120 mg every 8 weeks	9	Week 24: 12 (80)	Week 24: 7/15 (46.7)	Week 24: 3/15 (20)	NR	Patients withdrew during phase I	Interventional non-RCT
	LAN 120 mg every 6 weeks then every 4 weeks	13	Week 48: 10 (76.9)	Week 48: 3/13 (23.1)	Week 48: 2/13 (15.4)		NA	
	LAN 120 mg every 6 weeks^a^	70	Week 48: 49 (70)	Week 48: 32/70 (45.7)	Week 48: 4/70 (5.7)		Patients withdrew prematurely	
	LAN 120 mg every 6 weeks then 8 weeks	26	Week 48: 20 (76.9)	Week 48: 12/26 (46.2)	Week 48: 2/26 (7.7)		NA	
Schopohl 2011 [57]	LAN 120 mg every 4 weeks	17	One injection interval after the 6th injection: NR (82.4)	NR	NR	NR	Serious AEs were not reported by dosing regimen. None of the serious AEs were judged by the study investigator to be related to study treatment. However, the serious case of cholecystolithiasis was judged to be possibly related to study treatment by the treatment manufacturer	Interventional non-RCT
	LAN 120 mg every 6 weeks	12	One injection interval after the 6th injection: NR (83.3)					
	LAN 120 mg every 8 weeks	8	One injection interval after the 6th injection: NR (87.5)					
Lombardi 2009 [53]	LAN 120 mg every 4–8 weeks (not reported for individual treatment arms)	51	NR	NR	Week 48–52: 2/51 (NR)	NR	NA	Interventional non-RCT
*Studies assessing pegvisomant*								
Colao 2019 [40]	OCT 40 mg every 4 weeks	7	Month 8: 4 (57.1)	Month 8: 1/7 (14.3)	Month 8: 0/7 (NR)	NR	More patients in combination therapy arms than in the monotherapy group reported AEs with a suspected relationship to the study drug	RCT
	OCT 40 mg every 4 weeks + cabergoline 0.25–0.5 mg twice weekly, 4 × weekly then daily (from Week 4)	31	Month 8: 22 (71)	Month 8: 10/31 (32.3)	Month 8: 2/31 (6.5)			
	Pegvisomant 70 mg weekly + OCT 40 mg every 4 weeks	32	Month 8: (65.6)	Month 8: 15/32 (46.9)	Month 8: 1/32 (3.1)			
Madsen 2011 [43]	OCT 10–30 mg every 4 weeks or LAN 80 mg every 4 weeks	6	Week 24: 1 (NR)	NR	NR	NR	NA	RCT
	Pegvisomant 15–30 mg twice weekly + OCT or LAN at half usual dosage every 4 weeks^b^	12	Week 24: 3 (NR)					
van der Lely 2011 [48]	Pegvisomant 40–80 mg weekly to twice weekly + LAN 120 mg every 4 weeks	57	NR	Week 28: 24/57 (42)	Week 28: 8/57 (14)	NR	NA	Interventional non-RCT
Neggers 2009 [45]	Pegvisomant 20–200 mg weekly or twice weekly + OCT or LAN dose not reported^b^	86	NR	Mean follow-up 29.2 months: 1/86 (NR)	NR	NR	NA	Interventional non-RCT
Dassie 2019 [35]	Pegvisomant monotherapy 12 mg non-daily (some patients on unspecified combination therapy)	14	NR	Mean follow-up 81 months: 1/14 (NR)	NR	NR	Measured on last follow-up visit	Interventional non-RCT
PAPE (Muhammad 2018) [44]	Pegvisomant 61–134 mg weekly + LAN 120 mg or OCT 30 mg every 4 weeks^b^	61	NR	NR	Week 12: 3/61 (NR)	NR	NA	Interventional non-RCT
Neggers 2007 [47]	Pegvisomant 60 mg once or twice weekly (40 mg starting dose) + ﻿LAN 120 mg or OCT 30 mg every 4 weeks^b^	32	NR	NR	NR	NR	Patient with diabetes developed transient drug-induced hepatitis	Interventional non-RCT
PEGASO (Camara 2019) [39]	Pegvisomant non-daily (dosage and drug combination unclear)	108	NR	NR	NR	Unknown timepoint during pegvisomant regimen: 35/108 (83.3)	Adherence was indirectly determined using the Haynes-Sackett questionnaire. Adherence for non-daily pegvisomant regimens are significantly lower than daily regimens (61/108 [95.3%]; p = 0.048)	

Percentages are stated where reported. All timepoints represent the study end unless otherwise indicated; NR indicates results which were not reported

*AE* adverse event, *LAN* lanreotide autogel/depot, *NA* not applicable, *NR* not reported, *OCT* octreotide long-acting release, *RCT* randomized controlled trial, *SRL* somatostatin receptor ligand, *TRAE* treatment-related adverse event

^a^This was an initial phase where treatment was administered with 6-week EDIs for 24 weeks, after which patients were assigned to either 4-, 6- or 8-week intervals; the 15 patients reported here withdrew after this initial phase

^b^This study reported a combined result for patients receiving LAN or OCT as the concomitant treatment

Only a single study assessed treatment adherence with LAN administered at standard intervals versus EDIs, measured using the number of omitted doses as reported by patients, and found it to be higher with treatment at standard intervals (Table 2) [50]. An additional study with LAN found no significant difference in treatment adherence between patients treated at 5–6 and 7–8 week intervals (Table 2) [51].

#### Patient-reported outcomes

Patient-reported outcomes (PROs) were reported by eight studies that evaluated treatment with SRLs at EDIs [49–51, 53–55, 57, 58]; of these, seven reported on HRQoL, using the Acromegaly Quality of Life Questionnaire (AcroQoL), EQ-5D, Nottingham Health Profile Questionnaire, and treatment acceptance visual analog scale (VAS; Table 3) [49, 51, 53–55, 57, 58]. A single study assessing OCT at EDIs observed no decline in HRQoL (Nottingham Health Profile) with treatment at EDIs compared with baseline (before which, four-weekly dosing was used) [58]. In the remaining 6/7 studies, which administered LAN at EDIs, HRQoL was generally maintained or showed numerical improvement at study end compared with baseline, with no deterioration versus standard dosing [49, 51, 53–55, 57]. Patients’ treatment satisfaction was found to be high with EDIs in 2/2 studies, and higher with EDIs compared with standard intervals in 1/1 study [51, 54]. Patients preferred treatment at EDIs in 2/2 studies [55, 57]. One study reported that preference for treatment at EDIs was likely due to less frequent injections and visits to the physician possibly translating into a more convenient and comfortable therapy [57].

**Table 3:** Su﻿mmary of HRQoL and economic results

Study	Treatment	N	HRQoL instrument	HRQoL results		Measurement of patient satisfaction or preferences	Patient satisfaction and preferences		Economic results
*Studies assessing SRLs*									
Abrams 2007 [49]	LAN 60–120 mg every 4–6 weeks	9	4-point symptom scale (absent = 0, mild = 1, moderate = 2, severe = 3)	Baseline: 2.2 ± 2.2 Week 36: 1.0 ± 1.0 points		NR	NR		Injection costs per year (Belgium): decrease in cost from $181,940 to $137,077 (USD; converted from EUR and estimated on currency exchange rates of 2007)
	LAN 120 mg every 3 weeks	12	4-point symptom scale (absent = 0, mild = 1, moderate = 2, severe = 3)	NR		NR	NR		Injection costs per year (Belgium): increase in cost from $284,104 to $378,806 (USD) if dosing frequency increased from every 4 to every 3 weeks (converted from EUR and estimated on currency exchange rates of 2007)
SOMACROL (Bernabeu 2020) [51]	LAN 120 mg every > 4 weeks	109	AcroQol (range from 0 [worst possible HRQoL] to 100 [best possible HRQoL])	Month 6 or greater mean ± SD: 63 ± 20.1		Measured using the TSQM-9 treatment satisfaction questionnaire (scale 0–100, where 100 indicates highest treatment satisfaction)	Mean TSQM-9 treatment satisfaction questionnaire: 70.6 ± 18.7 for effectiveness, 69.1 ± 17.6 for convenience and 75.1 ± 16.6 for global satisfaction. 82.6% of patients preferred healthcare professional administration to self-administration, though they only received one or the other		NR
			EQ-5D	Month 6 or greater: 68.5% patients reported issues with pain or discomfort, 51.9% with anxiety/depression, 50.9% with mobility, 46.3% with daily activities, and 20.4% with self-care (20.4%). Mean (SD) EQ VAS score was 69.7 (17.9)					
LEAD (Neggers 2015) [55]	LAN 120 mg every 6 then every 4 weeks	9	AcroQol (range from 0 [worst possible HRQoL] to 100 [best possible HRQoL])	Baseline mean ± SD: 66.5 ± 15.9 Week 48 mean ± SE change from baseline: 2.0 ± 3.3		Patients were asked by the investigators to state their treatment preferences for LAN vs OCT at Week 48	76.9% of patients preferred LAN compared to OCT		NR
	LAN 120 mg every 6 weeks	55		Baseline mean ± SD: 61.4 ± 20.3 Week 48 mean ± SE change from baseline: − 1.3 ± 1.4			77.9% of patients preferred LAN compared to OCT		
	LAN 120 mg every 6 then 8 weeks	21		Baseline mean ± SD:59.5 ± 19.5 Week 48 mean ± SE change from baseline: − 1.9 ± 2.2			92.3% of patients preferred LAN compared to OCT		
Lombardi 2009 [53]	LAN 120 mg every 8 weeks then 4 weeks	19	Nottingham Questionnaire (higher score indicates worse QoL)	Baseline mean ± (SD): 6.7 ± 5.5 Week 52 mean ± (SD): 4.9 ± 5.8	Significant improvement in QoL observed across treatment groups (Wilcoxon signed rank: p < 0.001)	NR	NR		NR
	LAN 120 mg every 8 weeks then 6 weeks	15		Baseline mean ± (SD): 10 ± 9.8 Week 48 mean ± (SD): 6.6 ± 8.0					
	LAN 120 mg every 8 weeks	17		Baseline mean ± (SD): 11.1 ± 8.1 Week 48 mean ± (SD): 7.1 ± 6.3					
Lucas 2006 [54]	LAN 120 mg every 4 weeks	53	NR	NR		Treatment acceptance VAS score (range from 1 to 10, with 10 as maximum acceptance of treatment)	Mean (95% CI): 8.2 (7.0–9.3)		NR
	LAN 120 mg every 6 weeks	31		NR			Mean (95% CI): 8.4 (7.5–9.2)		
	LAN 120 mg every 8 weeks	13		NR			Mean (95% CI): 8.3 (95% CI 7.9–8.8)		
Schopohl 2011 [57]	LAN 120 mg every 8 weeks	7	AcroQol (range from 0 [worst possible HRQoL] to 100 [best possible HRQoL])	Study end mean ± SD (one injection interval after 6th injection): 49.8 ± 15.4		Patients were asked, via a questionnaire, whether they would prefer to receive the study treatment or their previous treatment in the future	57.1% of patients preferred LAN compared to monthly OCT. 14.3% of patients had no treatment preference	Study noted that for patients with adequately controlled disease at EDIs, less frequent injections and visits to the physician may translate to a more convenient and comfortable therapy, as reflected in the questionnaires	NR
	LAN 120 mg every 6 weeks	11		Study end mean ± SD (one injection interval after 6th injection): 54.8 ± 17.7			63.6% of patients preferred LAN compared to monthly OCT. 18.2% of patients had no treatment preference		
	LAN 120 mg every 4 weeks	17		Study end mean ± SD (one injection interval after 6th injection): 62.9 ± 18.2			11.8% of patients preferred LAN compared to monthly OCT. 41.2% of patients had no treatment preference		
Alvarez-Escola 2019 [50]	LAN 60 mg every 4 weeks	13	NR	NR		NR	NR		Days of absenteeism until disease control obtained, defined as the total sum of visits to doctor, hospital nurse or outpatient nurse: median (range): 10 (3–151)
	LAN 90 mg every 4 weeks	6							Days of absenteeism until disease control obtained, defined as the total sum of visits to doctor, hospital nurse or outpatient nurse: median (range): 8.5 (5–20)
	LAN 120 mg every 4 weeks	13							Days of absenteeism until disease control obtained, defined as the total sum of visits to doctor, hospital nurse or outpatient nurse: median (range): 13 (3–146)
	LAN 120 mg every 6 weeks	6							Days of absenteeism until disease control obtained, defined as the total sum of visits to doctor, hospital nurse or outpatient nurse: median (range): 9 (2–81)
	LAN 120 mg every 8 weeks	9							Days of absenteeism until disease control obtained, defined as the total sum of visits to doctor, hospital nurse or outpatient nurse: median (range): 6 (2–23)
Biermasz 2003 [58]	OCT 10–20 mg every 6 weeks	13	The Nottingham Health Profile range from 0 [no distress] to 100 [severe distress])	Week 26 mean ± SEM: 4 ± 1.4 Week 44 mean ± SEM: 4 ± 1.3		NR	NR		NR
Turner 2004 [61]	OCT 20–30 mg every 4 weeks (end dose)	2	NR	NR		NR	NR		Annual cost of OCT: $302,363 (USD; converted from GBP, estimated on currency exchange rates of 2003)
	OCT 20–30 mg every 6, 8, 10 and 12 weeks (end dose)	17							Annual cost of OCT (across all patients treated at EDIs): $162,674 (USD; final cost of 53.8% of a 4-weekly injection price of $302,363 [p < 0.01 vs 4-weekly dosing]; converted from GBP, estimated on currency exchange rates of 2003)
*Studies assessing pegvisomant*									
van der Lely 2011 [48]	Pegvisomant 40–80 mg weekly to twice weekly + LAN 120 mg every 4 weeks	57	AcroQol (range from 0 [worst possible HRQoL] to 100 [best possible HRQoL])	Week 28 change from baseline, mean ± SD: 2.2 ± 8.8		NR	NR		NR
Colao 2019 [40]	OCT 40 mg every 4 weeks	7	AcroQol (range from 0 [worst possible HRQoL] to 100 [best possible HRQoL])	Month 8 mean change from baseline: 2.27		NR	NR		NR
	OCT 40 mg every 4 weeks + cabergoline 0.25–0.5 mg twice weekly, 4 × weekly then daily (from Week 4)	32		Month 8 mean change from baseline: 2.58					
	Pegvisomant 70 mg weekly + OCT 40 mg every 4 weeks	31		Month 8 mean change from baseline: 1.24					
Higham 2009 [36]	Pegvisomant monotherapy 10–20 mg twice weekly	7	AcroQol (range from 0 [worst possible HRQoL] to 100 [best possible HRQoL])	Baseline mean ± SD: 81 ± 15 Week 0–16 mean ± SD: 83 ± 18		NR	NR		NR
	Pegvisomant monotherapy 10–20 mg weekly			Week 16–32 mean ± SD: 88 ± 15					
Madsen 2011 [43]	Pegvisomant 15–30 mg twice weekly + OCT or LAN at half usual dosage every 4 weeks	12	EQ-5D VAS	Baseline mean ± SEM: 68.2 ± 8.5 Week 24 mean ± SEM: 77.3 ± 4.6		NR	NR		NR
			PASQ (range from 0 [lowest symptom severity] to 40 [highest symptom severity])	Baseline median (range): 2 (0–8) Week 24 median (range): 2 (0–5)					
Neggers 2008 [46]	Pegvisomant 40 mg weekly + LAN or OCT	20	AcroQol (range from 0 [worst possible HRQoL] to 100 [best possible HRQoL])	Week 36 median change from baseline: 6.4 ± 4.25%		NR	NR		NR
			PASQ	Week 36 change from baseline: -2.0 ± 6.60					
Neggers 2007 [47]	Pegvisomant 60 mg once or twice weekly (40 mg starting dose) + LAN 120 mg or OCT 30 mg every 4 weeks	32	PASQ	Median follow-up 138 weeks improvement in QoL vs baseline: p < 0.05 (scores not reported)		NR	NR		NR
Bonert 2020 [38]	Pegvisomant 40–160 mg weekly + high dose LAN 120 mg or OCT 30 mg every 4 weeks	15	NR	NR		NR	NR		Total cost of treatment per year; $ (USD): $171,135 ± 19,745 (converted from monthly costs; not significant vs low dose SRL and weekly pegvisomant)
	Pegvisomant 40–160 mg weekly + low dose LAN 60 mg or OCT 10 mg every 4 weeks	23							Total cost of treatment per month; $ (USD): $118,038 ± 16,499 (converted from monthly costs; p < 0.05 vs low dose SRL + daily pegvisomant)
	Pegvisomant 15–60 mg daily + low dose LAN 60 mg or OCT 10 mg every 4 weeks	14							Total cost of treatment per month; $ (USD): $270,514 ± 133,901 (converted from monthly costs; p < 0.05 vs high dose SRL + weekly pegvisomant)

*AcroQoL* Acromegaly Quality of Life Questionnaire, *CI* confidence interval, *EDI* extended-dosing interval, *EUR* euro, *GBP* British pound sterling, *HRQoL* health-related quality of life, *LAN* lanreotide autogel, *NR* not reported, *OCT* octreotide long-acting release, *PASQ* Patient-Assessed Symptom Questionnaire, *QoL* quality of life, *SD* standard deviation, *SE* standard error, *SEM* standard error of the mean, *SRL* somatostatin receptor ligand, *TSQM-9* Treatment Satisfaction Questionnaire for Medication, *USD* United States dollar, *VAS* visual analog scale

#### Economic outcomes and healthcare resource use

Substantial cost savings ranging from $44,863–$146,667 [USD; converted from euros (EUR) and British pounds (GBP), respectively] per year were observed with the use of LAN or OCT at EDIs compared with standard dosing intervals, even with higher doses approved for use at EDIs (n = 2 studies [49, 61]; Table 3). Moreover, fewer days of absenteeism were associated with LAN treatment at EDIs as compared with standard dosing intervals (n = 1 study [50]).

### Pegvisomant

#### IGF-I

Figure 3c presents IGF-I results from studies assessing pegvisomant administered at EDIs. Among the two studies assessing pegvisomant monotherapy in which all patients had baseline IGF-I control, IGF-I levels decreased in one study and IGF-I control was maintained to study end in 73% of patients in the other [36, 37]. IGF-I levels also decreased from baseline with pegvisomant monotherapy in a further study which did not report on the proportion of patients with IGF-I control [35].

Studies investigating pegvisomant combination therapy tended to report on pegvisomant administered at EDIs (ranging from non-daily to weekly dosing and 30 to 134 mg in dosage) in combination with either LAN or OCT at standard dosing regimens (where reported, and administered at dosages of 120 mg and 10 to 30 mg, respectively). Where all patients had controlled IGF-I levels at baseline, IGF-I levels decreased by study end with pegvisomant combination therapy (n = 2 studies [43, 46]; pegvisomant dosing once to twice weekly; Fig. 3c). Where no patients had controlled IGF-I at baseline, IGF-I control was achieved by study end in ≥ 70% of patients in 4/5 studies [40, 41, 45, 47, 48].

#### Tumor size

A reduction in tumor volume of 20–25% was observed with pegvisomant combination therapy at EDIs in 13.2–19% of patients in 4/4 studies (Table 1). This shrinkage was considered clinically significant in three of these studies, but was considered most likely to be the effect of continued SRL therapy, and may have been maximal during prior SRL therapy at standard intervals [41, 45, 47, 48]. Two studies reported tumor growth in 1–24.5% of patients [41, 48]. In a single study that assessed tumor size following pegvisomant monotherapy at EDIs, neither tumor growth nor shrinkage was observed [37].

#### Safety and tolerability outcomes

Regarding treatment with pegvisomant combination therapy, AEs occurred in a similar proportion of patients treated at EDIs and standard dosing intervals (n = 2 studies reporting the total proportion of patients with AEs of any severity and organ class [40, 43]; Table 2). AEs were mostly mild or moderate in severity [40]. Serious AEs and serious TRAEs were experienced by a low proportion of patients across all EDI pegvisomant treatment regimens (n = 3 studies [40, 44, 48]; Table 2). No study assessing pegvisomant monotherapy reported AEs and no study examining pegvisomant at EDIs either as mono- or combination therapy reported on treatment-related deaths. Treatment adherence was higher at standard intervals compared with EDIs (n = 1 study), measured with the patient-reported Haynes-Sackett questionnaire, but it was unclear whether patients received pegvisomant combination therapy or monotherapy at the time the questionnaire was administered (Table 2) [39]. One patient-reported reason for lack of adherence to EDIs of pegvisomant was forgetfulness due to non-daily administration [39].

#### Patient-reported outcomes

For patients previously receiving pegvisomant monotherapy treatment daily, HRQoL scores improved from baseline with either twice weekly and weekly dosing, by study end (n = 1 study [36]; Table 3). Improvements in QoL scores from baseline to study end with pegvisomant combination therapy were observed with AcroQoL, Patient-Assessed Symptom Questionnaire (PASQ) and EQ VAS (n = 5 studies), with this result being clinically meaningful for EQ VAS in one study (+ 9.1; MCID = 8 [62]; Table 3 [40, 43, 46–48]).

#### Economic outcomes and healthcare resource use

Despite the cost per dose of pegvisomant being much lower than that of SRLs, a single study with pegvisomant combination therapy found a substantial annual cost saving of ~ $99,400–$152,500 (USD) when pegvisomant was administered weekly rather than daily (costs converted from monthly costs; Table 3) [38]. No identified studies assessed cost or healthcare resource use with pegvisomant monotherapy at EDIs.

### Quality and meta-analysis feasibility assessments (SRLs and pegvisomant)

Quality assessments, conducted using the AHFMR checklist for quantitative studies [33], revealed substantial variation in study quality, with full results presented in Table 4. A feasibility assessment determined that conducting any meta-analyses using data identified in this systematic literature review would not be appropriate due to the limited and heterogeneous nature of the evidence base. Specifically, patient numbers in the studies were generally small, and few relevant studies reported on each outcome with each treatment. Additionally, the variation in endpoints used (e.g., thresholds defining normal GH), timepoints of reporting, prior treatments, units of reporting GH and IGF-I (e.g., mU/L, xULN, and ng/mL), assays used for measuring GH and IGF-I, and levels of patient biochemical control at baseline precluded conducting a meaningful meta-analysis.

**Table 4:**
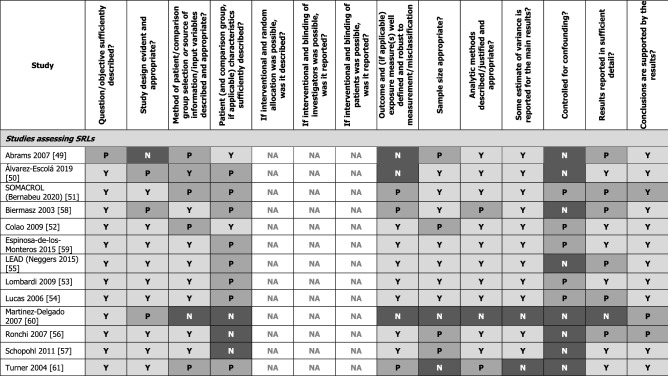

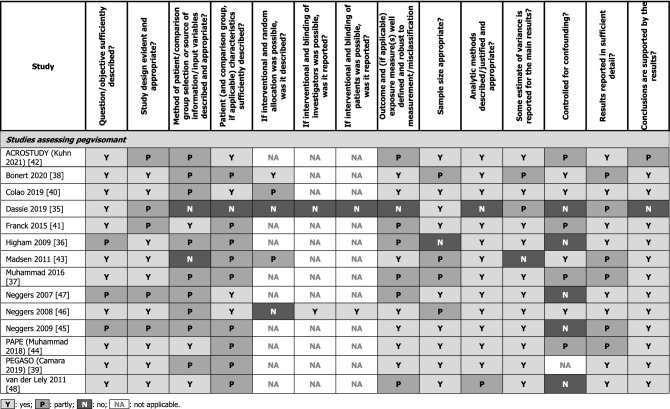
Summary of quality assessments

## Discussion

**Fig. 4:**
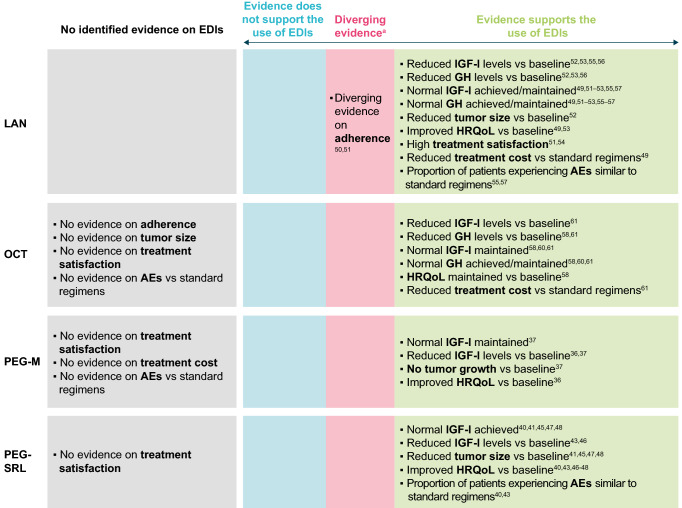
Overall conclusions regarding EDI use. ^a^Some evidence supports and some does not support the use of EDIs. Lower adherence was observed with EDIs of pegvisomant treatment compared to standard regimens, however it was unclear if this treatment was pegvisomant monotherapy or combination therapy [39]. *EDI* extended dosing interval, *GH* growth hormone, *HRQoL* health-related quality of life, *IGF-I* insulin-like growth factor I, *LAN* lanreotide autogel, *OCT* octreotide long-acting release, *PEG-M* pegvisomant monotherapy, *PEG-SRL* pegvisomant in combination with SRLs

This is the first known systematic literature review identifying evidence on EDIs for treatments in acromegaly, capturing data on clinical efficacy/effectiveness, safety and tolerability, PROs such as HRQoL, and economic outcomes (Fig. 4). Overall, the results of this systematic literature review suggest that EDIs of LAN, OCT, or pegvisomant in combination regimens may be effective treatment options for maintaining or achieving disease control. Only LAN has been licensed for use at EDIs of 6 to 8 weeks in Europe, the US, and some countries in Asia for patients whose acromegaly is stable with dosing every 4 weeks [11, 12, 21]. Conversely, OCT has not been licensed for treatment at EDIs, potentially explaining the limited evidence supporting less-frequent dosing regimens.

Consensus criteria for the diagnosis and management of acromegaly have been published [5, 6, 17]. In this systematic literature review, the diagnostic criteria for acromegaly and definitions of biochemical control used varied between studies and therefore did not always align with current consensus criteria. This heterogeneity likely reflects the observed variation in study year, design (observational versus interventional studies), geography, and consensus criteria, with biochemical cut-off values changing over time [5, 63, 64]. However, eligible articles were required to be published in peer-reviewed journals and/or congresses. Therefore, articles were of high quality, using appropriate definitions of biochemical control, with minimal risk that patients were misdiagnosed with acromegaly. If articles had been excluded because they did not use current consensus criteria, relevant data may have been missed.

In this systematic literature review, patients were commonly treated with LAN or OCT at EDIs following achievement of biochemical control in the included observational studies. Biochemical control was generally maintained in patients who had biochemically controlled acromegaly at baseline following treatment with LAN or OCT at EDIs [49, 55, 58, 60]. Treatment with SRLs at EDIs was also able to achieve disease control in some studies where patients did not have biochemically controlled disease at baseline [52, 53, 61]. These results suggest that using SRLs at EDIs may not only effectively maintain disease control, but have also been shown to achieve disease control in a proportion of patients who previously had uncontrolled disease without treatment.

Among observational studies assessing pegvisomant, no clear pattern emerged regarding why patients were treated at EDIs. Healthcare professionals may prescribe treatments at EDIs to reduce patient burden, or alternatively, based on economic rather than clinical considerations [39, 42]. Nearly all studies with pegvisomant at EDIs in combination with SRLs showed an increase from baseline in the proportion of patients who maintained or achieved disease control by study end [41, 45, 47, 48]. These results suggest that the addition of pegvisomant at EDIs may benefit patients whose disease is incompletely controlled with SRLs alone. Studies did not indicate when monotherapy or combination therapy would be more appropriate for patients, and further studies could assess whether patients with more severe disease may benefit from a combination regimen.

Definitions of biochemical control varied across studies and duration of prior biochemical control was often not reported, potentially impacting the interpretation of response to treatment at EDIs. The duration of prior treatment was infrequently reported, and study treatment varied across studies. However, patients were generally required to have received their prior treatment for ≥ 6 months to participate in included studies, and usually received the study treatment for ≥ 6 months across studies. Therefore, patients were likely to have reached a steady state prior to initiation of EDIs, and carry-over effects from prior treatment were likely to be small in the majority of studies. However, carry-over effects cannot be ruled out in studies in which durations of prior or study treatment were ≤ 6 months. Nonetheless, variation in the durations of prior and study treatment may reflect real-world practice, in which there is no required duration of treatment at standard intervals prior to initiation of EDIs, and provides evidence on outcomes with EDIs regardless of prior treatments and biochemical control.

Few studies (6/27) reported on tumor size change despite the clinical relevance of this outcome, most likely due to the non-interventional study design of many of the included articles. Nevertheless, results suggest that tumors continued to shrink once patients were switched from standard intervals to EDIs of LAN and/or pegvisomant combination therapy, with shrinkage being considered clinically significant in three studies reporting on pegvisomant in combination with SRLs [41, 45, 47, 48, 52]. The treatment goal of reducing tumor size [65] and the small number of studies that provided evidence on this outcome, particularly with treatment with SRLs, highlight the need for more research in this area.

A reduced risk of other TRAEs could be somewhat expected when dosing frequency is reduced [51]. This systematic literature review found that a comparable number of AEs and serious AEs were reported in patients treated at EDIs as compared to standard dosing regimens (reported by interventional studies only). This result, in part, may be explained by patients switching from standard to extended dosing regimens without a wash-out period [40, 43, 45, 48, 55, 57]. Consequently, AEs occurring following commencement of EDI regimens may have been related to the previous standard regimens. Alternatively, some AEs, such as gastrointestinal AEs, fade with continued SRL treatment [66]. Therefore, the incidence of AEs may have decreased while patients were receiving prior treatment with SRLs at standard intervals (prior treatment duration in these studies was ≥ 2 years [55, 57]), thus possibly explaining why no substantial difference in the incidence of AEs was observed between patients remaining on standard intervals and patients switching to EDIs. Additionally, the included studies may not have identified the true differences in the safety profiles of patients receiving standard dosing versus EDIs due to the limited number of patients for which data were reported. Similarly, the limited study durations may have precluded the observation of AEs which often have delayed onset, such as cholestasis [67]. While no specific data regarding the relationship between the occurrence or severity of comorbidities and treatment at EDIs were found, previous work has indicated that a number of comorbidities improve with disease control [68].

Lower treatment adherence to EDIs of LAN or pegvisomant, compared with standard dosing intervals [39, 50], may be due to patients on these regimens generally already having well-controlled disease, potentially causing them to overlook the importance of regular medication. Furthermore, EDIs at 6-week intervals would require dose administration at irregular points in the month, possibly making it easier to miss doses than with 4-week intervals, which are administered at approximately the same time each month. However, this systematic literature review found evidence on adherence to be largely inconclusive due to limited and diverging data and the use of self-reported methods to measure adherence (such as a patient-reported questionnaire), which are subjective and can overestimate adherence due to possible recall or reporting bias [69]. Although none of the identified studies in this systematic literature review investigated the use of oral treatments, adherence to treatment may be improved with the use of oral medications with straightforward dosing regimens (e.g., once daily with no fasting required) in place of injected medications such as SRLs and pegvisomant. Additionally, although no studies assessing persistence were identified, if patients find an EDI schedule less burdensome, persistence may be expected to increase.

No deterioration in HRQoL was observed with all treatments at EDIs compared with standard dosing intervals [36, 43, 46, 53–55, 57], and patients also tended to prefer LAN administered at EDIs over standard dosing regimens [55, 57]. This preference is likely due to the more convenient dosing schedules, less frequent travel to healthcare centers for receiving injections, and reduced costs to patients (including copayments). Indeed, previous work has shown that longer intervals between injections are less disruptive to patients’ lifestyles [70]. These results reflect the maintained clinical efficacy/effectiveness with treatment at EDIs as compared with treatment at standard regimens, and suggest that EDIs may help to reduce the life-long treatment burden experienced by patients with acromegaly [1, 55, 71, 72].

Although only a small number of studies reported on costs, the use of SRL monotherapy and pegvisomant in combination with SRLs at EDIs unsurprisingly resulted in substantial cost savings compared with treatment at standard dosing intervals, due to reduced resource use [38, 49, 61]. These cost savings reflect both the smaller amount of medication required and reduced therapy administration time needed from HCPs with treatment at EDIs, demonstrating that EDIs could also provide an economic benefit. Furthermore, treatment at EDIs, compared with standard intervals, may also provide indirect cost savings to patients with evidence supporting fewer interruptions to their employment [50].

In light of the recent pandemic, more attention has been drawn to limiting the exposure of vulnerable patients to environments, such as healthcare settings, where there may be greater risk of contracting infectious diseases [73]. Such patients may include those with acromegaly as the condition is often complicated by diabetes mellitus and hypertension, both of which put patients at an increased risk of morbidity in the event of infection with COVID-19 [74]. If healthcare resources become limited in future pandemics, they may need to be prioritized carefully. Additionally, if healthcare centers are not able to function at usual capacity, leading to appointments/services being delayed or canceled [75], treatment at EDIs may therefore be especially beneficial in this context, as it reduces the frequency of patient visits to their HCP and associated risk of infectious disease transmission.

The most notable limitation of the data identified in this systematic literature review was the heterogeneity observed across studies, including differences in study designs and variability in outcomes assessed. For example, there was considerable variation in duration of prior treatment at EDIs, duration of biochemical control prior to baseline, definitions of biochemical control, and study treatment duration, which may all affect the comparability between studies and interpretation of results as previously discussed. However, this heterogeneity likely reflects real-world practice and has allowed evidence to be identified in patients with varying treatment and disease history. Additionally, patient population sizes tended to be small, which is unsurprising considering the rare nature of acromegaly, but contributes to uncertainty in the interpretation of results. Finally, as with most systematic literature reviews, this review may be affected by publishing bias, as positive results are more likely to be published than negative results.

The strengths of this systematic literature review include the rigor and thoroughness of the article search and review process. A stringent methodology was followed as recommended by the University of York CRD and the Cochrane Collaboration [32]. Additionally, a substantial number of unique studies were identified (n = 27), conducted across several different countries, meaning the results of this systematic literature review are probably relevant to multiple geographical settings. Further evidence was identified for the use of three different pharmacological treatments at EDIs, representing a significant coverage of the principal classes of pharmacological therapies used in the clinical management of acromegaly. Due to the broad range of outcomes assessed, this systematic literature review has identified evidence gaps and areas for additional research that may further elucidate the benefits of treatment at EDIs for both patients and healthcare systems.

Overall, administration of treatment for acromegaly at EDIs in patients with control at standard intervals generally maintained at least the same level of efficacy/effectiveness as treatment with standard dosing intervals. Safety profiles for treatment at EDIs were similar to standard dosing, while evidence on adherence with EDI regimens was limited. Although limited data were reported regarding PROs, treatment at EDIs did not appear to cause any decrease in HRQoL, and patients favored treatment at EDIs as opposed to standard regimens. Limited data on economic outcomes demonstrate clear cost savings with administration of treatment at EDIs compared with standard dosing. Overall, evidence on EDI regimens indicate that efficacy/effectiveness, safety, and HRQoL can be maintained while potentially reducing the patient burden experienced with long-term treatment at standard dosing intervals. Considering the potential effectiveness of SRLs at EDIs for patients with good disease control, or the addition of pegvisomant at EDIs in combination with SRLs at standard intervals for those whose disease is not sufficiently controlled, physicians may wish to consider using EDIs. Further high-quality studies are required to determine the potential benefits of treatment at EDIs to patients and healthcare systems.

## Supplementary Information

Below is the link to the electronic supplementary material.

Supplementary file1 (PDF 551 KB)

## Data Availability

All data in this systematic literature review are from published literature and are included in this article.
