# Supplementary material for: A systematic literature review to evaluate extended dosing intervals in the pharmacological management of acromegaly
Source: Pituitary. 2022 Nov 29;26(1):9–41. doi: 10.1007/s11102-022-01285-1 (PMC9708130; doi:10.1007/s11102-022-01285-1)
Supplement: Supplementary file 1 — Supplementary file1 (PDF 551 KB) [file 11102_2022_1285_MOESM1_ESM.pdf]

A Systematic Literature Review to Evaluate Extended Dosing Intervals in the Pharmacological Management of Acromegaly

M. Fleseriu,[1] Z. Zhang,[2] K. Hanman,[3] K. Haria,[3] A. Houchard,[4] S. Khawaja,[5] A. Ribeiro-Oliveira Jr,[6] M. Gadelha[7]

[1]Pituitary Center at Oregon Health & Science University, Portland, OR, USA; [2]Department of Endocrinology and Metabolism, Huashan Hospital, Shanghai Medical College, Fudan University, Shanghai, China; [3]Costello Medical, London, UK; [4]Ipsen Pharma, Boulogne-Billancourt, France; [5]World Alliance of Pituitary Organizations, Zeeland, The Netherlands; [6]Ipsen, Cambridge, MA, USA; [7]Neuroendocrinology Research Center/Endocrinology Division, Medical School and Hospital Universitário Clementino Fraga Filho, Universidade Federal do Rio de Janeiro, Rio de Janeiro, Brazil

Correspondence to: Dr Maria Fleseriu, fleseriu@ohsu.edu

SUPPLEMENTARY DATA

Supplementary Table 1 Search Terms for Ovid MEDLINE and Epub Ahead of Print, In-Progress, In-Data-Review and Other Non-Indexed Citations and Daily

Term Group	#	Search Terms	Hits (30th June 2021)
**Acromegaly**	1	exp acromegaly/	8683
	2	acromegal$.ti,ab,kf.	9649
	3	1 or 2	11355
**Treatments**	4	(Somatuline$ or lanreotide$ or Sandostatin$ or octreotide$ or Dostinex$ or cabergoline$ or Signifor$ or pasireotide$ or Somavert$ or pegvisomant$ or Mycapssa$).ti,ab,kf.	11038
**Combined**	5	3 and 4	1609
**Exclusion terms**	6	exp animals/ not exp humans/	4853923
	7	(comment or editorial or historical article).pt.	1656116
	8	(case stud$ or case report$).ti.	321735
	9	historical article/	364229
	10	or/6-9	6768909
**Combine**	11	5 not 10	1515
	12	limit 11 to yr=2001-2021	1073

Supplementary Table 2 Search Terms for Embase

Term Group	#	Search Terms	Hits (30th June 2021)
**Acromegaly**	1	exp acromegaly/	12497
	2	acromegal$.ti,ab,kw.	11407
	3	1 or 2	14089
**Treatments**	4	(Somatuline$ or lanreotide$ or Sandostatin$ or octreotide$ or Dostinex$ or cabergoline$ or Signifor$ or pasireotide$ or Somavert$ or pegvisomant$ or Mycapssa$).ti,ab,kw.	17325
**Combined**	5	3 and 4	2393
**Exclusion terms**	6	(conference abstract or conference review).pt.	4132358
	7	limit 6 to yr=1948-2017	3019602
	8	exp animals/ not exp humans/	4801066
	9	(comment or editorial or historical article).pt.	695064
	10	(case stud$ or case report$).ti.	393298
	11	historical article/	1
	12	or/7-11	8582906
**Combine**	13	5 not 12	1901
	14	limit 13 to yr=2001-2021	1364

Supplementary Table 3 Combined Search Terms for Cochrane Database of Systematic Reviews (CDSR) and Cochrane Central Register of Controlled Trials (CENTRAL) [Searched Separately via Wiley Online Platform]

Term Group	#	Search Terms	Hits (25th June 2021)
**Acromegaly**	1	[mh "acromegaly"]	234
	2	acromegal*:ti,ab,kw	490
	3	#1 OR #2	490
**Treatments**	4	(Somatuline* OR lanreotide* OR Sandostatin* OR octreotide* OR Dostinex* OR cabergoline* OR Signifor* OR pasireotide* OR Somavert* OR pegvisomant* OR Mycapssa*):ti,ab,kw	2316
**Combined**	5	#3 AND #4	295
**CENTRAL**	6	#5 with Publication Year from 2001 to 2021, in Trials	228
**CDSR**	7	#5 with Publication Year from Jan 2001 to May 2021, in Cochrane Reviews and Cochrane Protocols	0

Supplementary Table 4 Search Terms for the Congress Searches

Conference	Search terms	Number of hits	Number of included abstracts
Endocrine Society Annual Meeting (ENDO)	• acromegaly<br>• acromegalic<br>• Somatuline<br>• Lanreotide<br>• Sandostatin<br>• octreotide<br>• Dostinex<br>• Cabergoline<br>• Signifor<br>• Pasireotide<br>• Somavert<br>• pegvisomant<br>• Mycapssa	• 2018: 217<br>• 2019: 151<br>• 2021: 260	• 2018: 1<br>• 2019: 0<br>• 2021: 0
International Congress of Endocrinology (ICE)		• 2018: 24<br>• 2021: 16	• 2018: 0<br>• 2021: 0
American Association of Clinical Endocrinologists (AACE) Annual Meeting		• 2019: 7<br>• 2020: 5<br>• 2021: 18	• 2019: 0<br>• 2020: 0<br>• 2021: 0
European Neuroendocrine Association (ENEA)		• 2018: 40<br>• 2020: 30	• 2018: 0<br>• 2020: 0
European Congress of Endocrinology (ECE)		• 2019: 174<br>• 2020: 162<br>• 2021: 107	• 2019: 1<br>• 2020: 0<br>• 2021: 0

Abbreviations: AACE: American Association of Clinical Endocrinologists Annual Congress; ECE: European Congress of Endocrinology; ENDO: Endocrine Society Annual Meeting; ENEA: European Neuroendocrine Association Congress; ICE: International Congress of Endocrinology.

Supplementary Table 5 Full Eligibility Criteria for the SLR

Domain	Inclusion criteria	Exclusion criteria
**Population**	Adult patients with acromegaly	Mixed cohorts of patients (e.g., with acromegaly and NETs) where results are not presented separately for patients with acromegaly
**Intervention**	The following treatments for acromegaly, administered as monotherapies or in combination, with extended dosing intervals as follows:<br>• Lanreotide® (Somatuline Autogel®)[a] administered at intervals of >4 weeks<br>• Octreotide injection (Sandostatin® LAR® Depot) administered at intervals of >4 weeks<br>• Oral octreotide (MYCAPSSA®) administered less often than twice daily<br>• Pasireotide (Signifor®) administered at intervals of >4 weeks<br>• Cabergoline (Dostinex®) administered less often than twice per week<br>• Pegvisomant (Somavert®) administered less often than once daily	• Octreotide implants<br>• Any other intervention (e.g., implants)<br>• Any listed intervention administered at standard or shorter dosing intervals
**Comparators**	• The same treatment at standard or shorter dosing intervals<br>• Any other treatment<br>• No comparator or placebo	NA

**Outcomes**	Relevant outcomes must be reported.<br>• Clinical outcomes:<br>GH<br>IGF-I<br>Tumor volume<br>• Safety and tolerability outcomes:<br>AEs<br>Serious AEs<br>Discontinuation<br>Adherence<br>• PROs:<br>QoL<br>Patient preferences and satisfaction<br>• Economic outcomes:<br>Costs<br>HCRU	• Any other outcomes
**Study design**	Any longitudinal study design:<br>• Interventional studies:<br>RCTs<br>Non-randomized trials<br>Single-arm interventional studies<br>• Observational studies:<br>Prospective studies<br>Retrospective studies	• Any other study types, including:<br>Case studies<br>Narrative reviews<br>Comments<br>Editorials<br>Modelling studies<br>SLRs or MAs[b]
**Publication type**	• Peer-reviewed publications published from 2001 onwards<br>• Conference abstracts published from 2018 onwards	• Peer-reviewed publications published up to 2000<br>• Conference abstracts published up to 2017
**Other considerations**	• Journal articles and conference abstracts in the English language only	• Non-English journal articles or conference abstracts

[a]Also known as Somatuline® Depot. [b]SLRs and MAs were hand-searched for relevant articles and then excluded at full-text review. **Abbreviations**: AE: adverse event; GH: growth hormone; HCRU: healthcare resource utilization; IGF-I: insulin-like growth factor I; LAR: long-acting release; MA: meta-analysis; NA: not applicable; NET: neuroendocrine tumor; PRO: patient-reported outcome; QoL: quality of life; RCT: randomized controlled trial; SLR: systematic literature review.

Supplementary Table 6 List of Data Extracted

Category	Information extracted
**Study characteristics**	• Study design<br>• Primary objective of study<br>• Study setting<br>• Study location<br>• Patient population size<br>• Treatment arms (dose and interval)
**Patient characteristics**	For each EDI treatment arm:<br>• Number of patients<br>• Sex<br>• Age<br>• Time since diagnosis<br>• Previous treatment<br>• Baseline IGF-I and GH levels<br>• Time since diagnosis<br>• Time with controlled IGF-I prior to EDI initiation<br>• Immunohistochemistry[a]
**Clinical outcomes**	For each EDI treatment arm:<br>• IGF-I levels<br>• GH levels<br>• Achievement of biochemical control<br>• Tumor size
**Safety and tolerability outcomes**	For each EDI treatment arm:<br>• AEs<br>• Adherence
**PROs**	For each EDI treatment arm:<br>• HRQoL<br>• Patient satisfaction and preferences
**Economic outcomes**	For each EDI treatment arm:<br>• Costs<br>• Resource use

[a]No included study reported on immunohistochemistry. **Abbreviations:** AE: adverse event; EDI: extended dosing intervals; GH: growth hormone; HRQoL: health-related quality of life; IGF-I: insulin-like growth factor I; PROs: patient-reported outcomes.

Supplementary Table 7 List of Deprioritized Studies

Study	Reason for exclusion
**Franck 2017 [11]**	Unclear if greater than five patients were treated at EDIs
**Jehle 2005 [14]**	Five or fewer patients treated at EDIs
**Khairi 2017 [15]**	Five or fewer patients treated at EDIs
**Lasolle 2019 [17]**	Five or fewer patients treated at EDIs
**Neggers 2014 [25]**	Five or fewer patients treated at EDIs
**Ramirez 2012 [29]**	Data on treatment at EDIs only available at baseline
**Sagvand 2016 [31]**	Five or fewer patients treated at EDIs
**Sesmilo 2014 [33]**	Five or fewer patients treated at EDIs
**Vilar 2014 [36]**	Data on treatment at EDIs only available at baseline

Abbreviations: EDIs: extended dosing intervals.

Supplementary Table 8 Summary of Study Characteristics

Study	Design	Primary objective of the study	Setting	Location	N (total)	Treatment arms
Studies assessing SRLs						
Abrams 2007 [1]	Interventional non-RCT	Investigate whether prolonging or shortening the interval between LAN would offer any benefit	NR	NR	21	Dose extension study:[a]<br>• LAN EDI 60–120 mg every 4–6 weeks (titrated)<br>• LAN non-EDI 120 mg every 3 weeks
Álvarez-Escolá 2019 [2]	Retrospective cohort	Determine the time to achieve normalization of GH and IGF-I levels in responding patients with acromegaly administered different dosage regimens of LAN	Single-country	Spain	57	• LAN 60 mg every 4 weeks<br>• LAN 90 mg every 4 weeks<br>• LAN 120 mg every 4 weeks<br>• LAN 120 mg every 6 weeks<br>• LAN 120 mg every 8 weeks
Biermasz 2003 [4]	Interventional non-RCT	Assess whether the dose interval could be safely increased from 4 to 6 weeks, without significant effect on serum GH concentrations or other biochemical and clinical markers of GH hypersecretion	Single-country	The Netherlands	14	• Withdrawal phase: no treatment<br>• OCT 10–20 mg every 6 weeks
Colao 2009 [7]	Interventional non-RCT	Evaluate GH and IGF-I control and tumor shrinkage in newly diagnosed patients with acromegaly treated first-line with LAN (autogel) 120 mg	Single-country	Italy	26	Dose extension study:[a]<br>• LAN 120 mg every 4 weeks<br>• LAN 120 mg every 6 weeks<br>• LAN 120 mg every 8 weeks
Espinosa-de-los-Monteros 2015 [10]	Retrospective cohort	Report our day-to day experience with the long-term use of OCT in the treatment of acromegaly	Single-country	Mexico	157	Dose extension study:[a]<br>• OCT 20 mg every 4–12 weeks
LEAD (Neggers 2015) [26]	Interventional non-RCT	Evaluate EDIs with LAN 120 mg in patients with acromegaly previously biochemically controlled with OCT 10 or 20 mg	Multi-country	Brazil, Denmark, Finland, France, Greece, Latvia, The Netherlands, Norway, Poland, Romania, Russia, Serbia, South Korea, and Sweden	124	• Phase I: LAN 120 mg every 6 weeks<br>• Phase II: LAN 120 mg every 4 weeks<br>• Phase II: LAN 120 mg every 6 weeks<br>• Phase III: LAN 120 mg every 8 weeks
Lombardi 2009 [18]	Interventional non-RCT	Evaluate efficacy and safety of LAN (autogel) 120 mg injections every 4–8 weeks in	Single-country	Italy	51	• LAN 120 mg every 8 weeks

Study	Design	Primary objective of the study	Setting	Location	N (total)	Treatment arms
		somatostatin analogue-naïve patients with acromegaly				• LAN 120 mg every 8 weeks then 6 weeks<br>• LAN 120 mg every 8 weeks then 4 weeks
Lucas 2006 [19]	Interventional non-RCT	Evaluate whether the dosing interval of LAN could be extended beyond 4 weeks without compromising efficacy or safety.	Multi-country	Portugal, Spain	97	• LAN 120 mg every 4 weeks<br>• LAN 120 mg every 6 weeks<br>• LAN 120 mg every 8 weeks
Martinez-Delgado 2007 [21]	Interventional non-RCT	Individualize the dose and frequency of OCT in acromegaly, including the minimum number of injections to maintain safe levels of GH and IGF-I	NR	NR	12	Dose extension study:[a]<br>• OCT 20 mg every 4 weeks<br>• OCT 20 mg every 8 weeks<br>• OCT 20 mg every 12 weeks
Ronchi 2007 [30]	Interventional non-RCT	Compare efficacy and tolerability of autogel 120 mg given every 4–8 weeks with those of OCT given every 4 weeks	Single-country	Italy	23	• LAN 120 mg every 4 weeks<br>• LAN 120 mg every 6 weeks<br>• LAN 120 mg every 8 weeks
Schopohl 2011 [32]	Interventional non-RCT	Assess the efficacy and safety of longer dosing intervals of LAN, Somatuline autogel (LAN), 120 mg in patients with acromegaly, previously treated with OCT	Single-country	Germany	37	• LAN 120 mg every 4 weeks<br>• LAN 120 mg every 6 weeks<br>• LAN 120 mg every 8 weeks
SOMACROL (Bernabeu 2020) [3]	Cross-sectional	Determine the effectiveness, as measured by IGF-I levels, of LAN 120 mg administered at dosing intervals >4 weeks for >6 months in patients with acromegaly treated in routine clinical practice	Multi-country	Portugal, Spain	109	• LAN 120 mg every 5–6 weeks<br>• LAN 120 mg every 7–8 weeks<br>• LAN 120 mg every >8 weeks
Turner 2004 [34]	Interventional non-RCT	Perform a prospective systematic study to determine whether extending the interval between doses of LAR allows maintenance of 'safe' GH in selected patients with acromegaly	NR	NR	22	Dose extension study:[a]<br>• OCT 20–30 mg every 4 weeks<br>• OCT 20–30 mg every 6 weeks<br>• OCT 20 mg every 8 weeks<br>• OCT 20–30 mg every 10 weeks<br>• OCT 20–30 mg every 12 weeks
Studies assessing pegvisomant						
ACROSTUDY (Kuhn 2021) [16]	Retrospective cohort	Define the medical reasons for the treatment of patients with pegvisomant as monotherapy (M) or combined with SA, either as primary bitherapy (PB) (pegvisomant is secondarily introduced after SA) or as secondary bitherapy	Single-country	France	312	• Pegvisomant monotherapy 15 mg, 1–14 injections weekly<br>• Pegvisomant primary bitherapy 10 mg, 1–14 injections weekly

Study	Design	Primary objective of the study	Setting	Location	N (total)	Treatment arms
		(SB) (SAs secondarily introduced after pegvisomant)				
Bonert 2020 [5]	RCT	Compare cost-effectiveness and efficacy of 3 lower-dose combination regimens in controlled and uncontrolled acromegaly	Single-country	USA	62	• Pegvisomant weekly 40–160 mg + LAN 120 mg or OCT 30 mg every 4 weeks<br>• Pegvisomant weekly 40–160 mg + LAN 60 mg or OCT 10 mg every 4 weeks<br>• High dose OCT or LAN + pegvisomant 40–160 mg weekly
Colao 2019 [8]	Mixed (RCT and non-RCT phases)	Investigate the efficacy and safety of high-dose Sandostatin® LAR® as monotherapy or in combination with pegvisomant (GH-receptor antagonist) or cabergoline (dopamine agonist) in a large population of acromegalic patients with pituitary adenomas following previous failure of first-generation SSA as a conventional SSA treatment	Multi-country	NR	70	• Baseline to Month 3: OCT 40 mg every 4 weeks<br>• Months 3–8: Pegvisomant 70 mg weekly + OCT 40 mg every 4 weeks
Dassie 2019 [9]	NR	Compare acromegalic patients on daily pegvisomant administration with patients on non-daily pegvisomant administration	NR	NR	43	• Pegvisomant monotherapy 12 mg non-daily<br>• Pegvisomant monotherapy 24 mg daily
Franck 2015 [12]	Interventional non-RCT	Assess the influence of d3-GHR on IGF-I levels and pegvisomant responsiveness in patients with acromegaly using combined pegvisomant and long-acting somatostatin receptor ligand (LA-SRIF) treatment	Single-country	The Netherlands	112	• Pegvisomant 80 mg weekly + OCT 30 mg or LAN 120 mg every month
Higham 2009 [13]	Interventional non-RCT	Determine the efficacy of weekly dosing of pegvisomant	Single-country	UK	7	• Pegvisomant monotherapy 10–20 mg twice weekly<br>• Pegvisomant monotherapy 10–20 mg weekly
Madsen 2011 [20]	RCT	Study whether patients sufficiently controlled on SA monotherapy can be transferred to combination therapy with low-dose pegvisomant and a reduced SA dose	Single-country	Denmark	18	• Pegvisomant twice weekly 30–60 mg + LAN 24 – 60 mg or OCT 6.7–20 mg every 4 weeks
Muhammad 2016 [23]	Prospective pilot study	Assess the efficacy of switching to pegvisomant monotherapy in patients well controlled on combination therapy of LA-SSAs and pegvisomant	NR	NR	15	• Pegvisomant monotherapy 60 mg weekly (starting dose) once or twice weekly
Neggers 2007 [28]	Interventional non-RCT	Assess long-term efficacy and safety in a larger group of acromegalic patients after a period of 138 (35-149) weeks [median (range)]	Single-country	The Netherlands	32	• Pegvisomant 60 mg once or twice weekly (40 mg starting dose) + LAN 120 mg or OCT 30 mg every 4 weeks
Neggers 2008 [27]	RCT	Assess whether weekly administration of pegvisomant improves QoL and metabolic parameters in acromegalic patients with normal	Single-country	NR	20	• Pegvisomant 40 mg weekly + LAN or OCT

Study	Design	Primary objective of the study	Setting	Location	N (total)	Treatment arms
		age-adjusted IGF-I concentrations during long acting SSA treatment				
Neggers 2009 [24]	Interventional non-RCT	Assess the long-term safety in a larger group of acromegalic patients over a larger period of time: 29.2 months	Single-country	The Netherlands	86	• Pegvisomant 20–200 mg weekly or twice weekly + OCT or LAN dose not reported
PAPE (Muhammad 2018) [22]	Interventional non-RCT	Assess the efficacy and safety of pasireotide long-acting release (PAS-LAR) alone or in combination with pegvisomant by switching patients with acromegaly who were well controlled with long-acting somatostatin analogues (LA-SSAs) and pegvisomant to PAS-LAR with or without pegvisomant	Single-country	NR	61	• Pegvisomant 61–134 mg weekly + LAN 120 mg or OCT 30 mg every 4 weeks
PEGASO (Camara 2019) [6]	Cross-sectional	Determine the adherence to pegvisomant treatment in patients with acromegaly in the real-world clinical practice setting in Spain.	Single-country	Spain	108	• Pegvisomant non-daily (dosage and drug combination unclear)
van der Lely 2011 [35]	Interventional non-RCT	Evaluate the efficacy and safety of co-administered LAN (120 mg/month) and pegvisomant (40-120 mg/week) in acromegaly	NR	NR	57	• Pegvisomant 40 mg weekly + LAN 120 mg every 4 weeks<br>• Pegvisomant 60 mg weekly + LAN 120 mg every 4 weeks<br>• Pegvisomant 80 mg weekly + LAN 120 mg every 4 weeks<br>• Pegvisomant 40 mg twice weekly + LAN 120 mg every 4 weeks<br>• Pegvisomant 60 mg twice weekly + LAN 120 mg every 4 weeks

Footnotes: [a]In these studies, patients all started on the same dosing regimen, and then dosing intervals were systematically altered based on IGF-I and/or GH levels (e.g., intervals were extended if biochemical control was achieved/maintained).

Abbreviations: d3-GHR: exon 3 deletion polymorphism of the growth hormone receptor; EDI: extended dosing interval; GH: growth hormone; IGF-I: insulin-like growth factor I; LAN: lanreotide autogel/depot; LAR: long-acting release; LA-SSA: long-acting somatostatin analogue; LA-SRIF: long-acting somatostatin receptor ligand; m: monotherapy; NR: not reported; OCT: octreotide long-acting release; PAS-LAR: pasireotide long-acting release; PB: primary bitherapy; QoL: quality of life; RCT: randomized controlled trial; SB: secondary bitherapy; SRL: somatostatin receptor ligand; SSA/SA: somatostatin analog; USA: United States of America.

Supplementary Table 9 Summary of Baseline Characteristics by Dosing Regimen

Study	Treatment	N	Male, N (%)	Age, years, mean (SD)	Age, years, median (range)	Time since diagnosis	Previous surgery, N (%)	Previous RT, N (%)	Previous treatments, N (%)	GH level, µg/L, mean (SD)	GH level, µg/L, median (range)	IGF-I level, mean (SD)	IGF-I level, median (range)	Time with controlled IGF-I prior to EDI initiation
Studies assessing SRLs														
Abrams 2007 [1]	LAN 60–120 mg every 4–6 weeks	9	5 (NR)	54.6 (13.1)	NR	Mean (SD): 10.4 (5.0) years	2 (NR)	1	SRL at equivalent weekly mean (SD) dose of 23.3 (7.0) mg: 9 (100) for a mean (SD) of 33.9 (21.1) months	1.4 (1.6)	NR	195 (68) µg/L	NR	NR
	LAN 120 mg every 3 weeks	12	6 (NR)	49.2 (16.2)	NR	Mean (SD): 7.3 (4.0) years	8 (NR)	3	SRL at equivalent weekly dose of 30 mg: 12 (100) for a mean (SD) of 23 (10.1) months	3.3 (1.6)	NR	444 (220) µg/L	NR	NR
Álvarez-Escolá 2019 [2]	LAN 60 mg every 4 weeks	13	4 (30.8)	NR	67 (27–83)	Median (range): 100.7 (14.6–285.0) months	8 (61.5)	2 (15.4)	LAN: 13 (100) for ≥4 months	NR	2.65 (0.2–20.5)	NR	1.6 (1.1–3.2) ×ULN	NA (no patients had biochemical control at baseline)
	LAN 90 mg every 4 weeks	6	1 (16.7)	NR	51.5 (30–77)	Median (range): 103.7 (8.1–128.4) months	5 (83.3)	2 (33.3)	LAN: 6 (100) for ≥4 months	NR	3.9 (3.2–48.2)	NR	4 (1.1–4.4) ×ULN	
	LAN 120 mg every 4 weeks	13	6 (46.2)	NR	57 (32–88)	Median (range): 129.9 (19.3–284.2) months	7 (53.8)	6 (46.2)	LAN: 13 (100) for ≥4 months	NR	2.52 (1.1–37.5)	NR	1.4 (1.0–3.4) ×ULN	
	LAN 120 mg every 6 weeks	6	3 (50)	NR	73 (23–90)	Median (range): 170.1 (17.9–325.5) months	4 (66.7)	1 (16.7)	LAN: 6 (100) for ≥4 months	NR	1.58 (0.5–3.7)	NR	1.1 (1.0–1.9) ×ULN	
	LAN 120 mg every 8 weeks	9	3 (33.3)	NR	70 (37–83)	Median (range): 120.8 (41.6–	7 (77.8)	1 (11.1)	LAN: 9 (100) for ≥4 months	NR	2.84 (0.9–8.0)	NR	1.4 (1.0–2.2) ×ULN	

Study	Treatment	N	Male, N (%)	Age, years, mean (SD)	Age, years, median (range)	Time since diagnosis	Previous surgery, N (%)	Previous RT, N (%)	Previous treatments, N (%)	GH level, µg/L, mean (SD)	GH level, µg/L, median (range)	IGF-I level, mean (SD)	IGF-I level, median (range)	Time with controlled IGF-I prior to EDI initiation
						218.6) months								
SOMACROL (Bernabeu 2020) [3]	LAN 120 mg every >4 weeks	109	47 (43.1)	59.1 (13.2)	NR	Mean (SD): 12.3 (9.6) years	53 (48.6)	5 (4.6)	• Lanreotide (other formulations) at mean (SD) dose of 68.6 (37.6) mg: 7 (6.4)<br>• OCT at mean (SD) dose of 31.1 (30.2) mg: 28 (25.7)<br>• Cabergoline: 6 (5.5)<br>• Pegvisomant: 1 (0.9)<br>• Bromocriptine: 1 (0.9)	NR	NR	NR	NR	NR
Biermasz 2003 [4]	Withdrawal phase (no treatment) followed by OCT 10–20 mg every 6 weeks	14	7 (NR)	58.3 (NR)	NR	NR	11 (NR)	4 (NR)	OCT 10–20 mg every 4 weeks: 14 (100) for 3–24 months (range)	NR	NR	NR	NR	NR
Colao 2009 [7]	LAN 120 mg every 8 weeks	9	3 (NR)	NR	NR (43–69)	NR (newly diagnosed patients)	NR	NR	None (study treatment was first-line)	NR	NR (1.3–23.9)	NR	NR (1.24–3.04) xULN	NA (newly diagnosed active acromegaly)
	LAN 120 mg every 6 weeks	8	2 (NR)	NR	NR (31–67)	NR (newly diagnosed patients)	NR	NR	None (study treatment was first-line)	NR	NR (8.0–75.0)	NR	NR (1.47–8.12) xULN	NA (newly diagnosed active acromegaly)
	LAN 120 mg every 4 weeks	9	4 (NR)	NR	NR (33–70)	NR (newly diagnosed patients)	NR	NR	None (study treatment was first-line)	NR	NR (1.5–58.4)	NR	NR (1.39–6.75) xULN	NA (newly diagnosed active acromegaly)
Espinosa-de-los-Monteros 2015 [10]	OCT 20 mg every 4–12 weeks	36[a]	NR	NR	NR	NR (diagnosed between 2003–2012)	NR	NR	OCT: 36 (100) for ≥6 months	NR	NR	NR	NR	NR

Study	Treatment		N	Male, N (%)	Age, years, mean (SD)	Age, years, median (range)	Time since diagnosis	Previous surgery, N (%)	Previous RT, N (%)	Previous treatments, N (%)	GH level, µg/L, mean (SD)	GH level, µg/L, median (range)	IGF-I level, mean (SD)	IGF-I level, median (range)	Time with controlled IGF-I prior to EDI initiation
LEAD (Neggers 2015) [26]	Phase 1	LAN 120 mg every 6 weeks	15	6 (40)	55.2 (15.3)	NR	Mean (SD): 6.9 (5.3) years	10 (NR)	NR	• OCT 10 mg every 4 weeks: 4 (28.6)<br>• OCT 20 mg every 4 weeks: 10 (71.4)<br>Mean (SD) duration: 2.2 (2.2) years	1.0 (1.0)	NR	93.3% (76.7) xULN	NR	NR
	Phase 2	LAN 120 mg every 4 weeks	13	5 (38.5)	55 (10.1)	NR	Mean (SD): 10.2 (5.9) years	10 (NR)	NR	OCT 20 mg every 4 weeks: 13 (100) for a mean (SD) of 2.5 (2.4) years	0.9 (0.7)	NR	98.7% (14.6) xULN	NR	NR
		LAN 120 mg every 6 weeks	70	29 (41.4)	53.2 (10.4)	NR	Mean (SD): 8.2 (5.3) years	54 (NR)	NR	• OCT 10 mg every 4 weeks: 11 (15.7)<br>• OCT 20 mg every 4 weeks: 59 (84.3)<br>Mean (SD) duration: 2.6 (2.4) years	0.9 (1.2)	NR	67.7% (29.6) xULN	NR	NR
		LAN 120 mg every 8 weeks	26	6 (23.1)	57 (9.8)	NR	Mean (SD): 10.2 (7.4) years	24 (NR)	NR	• OCT 10 mg every 4 weeks: 9 (34.6)<br>• OCT 20 mg every 4 weeks: 17 (65.4)<br>Mean (SD) duration: 2.5 (1.8) years	1.1 (1.2)	NR	52.5% (25.0) xULN	NR	NR
Lombardi 2009 [18]	LAN 120 mg every 8 weeks		17	7 (NR)	59 (12)	NR	NR	4 (NR)	NR	SRL-naïve and dopamine agonist-naïve: 17 (100)	9.2 (12.6)	NR	594 (279) µg/L	NR	NA (no patients had biochemical control at baseline)
	LAN 120 mg every 8 weeks then 6 weeks		15	7 (NR)	52 (13)	NR	NR	3 (NR)	NR	SRL-naïve and dopamine agonist-naïve: 15 (100)	16.9 (19)	NR	635 (178) µg/L	NR	NA (no patients had biochemical control at baseline)
	LAN 120 mg every 8 weeks then 4 weeks		19	9 (NR)	39 (11)	NR	NR	5 (NR)	NR	SRL-naïve and dopamine agonist-naïve: 19 (100)	30.5 (24.7)	NR	855 (275) µg/L	NR	NA (no patients had biochemical control at baseline)
Lucas 2006 [19]	LAN 120 mg every 4–8 weeks		97	44	50.6 (13.6)	NR	Mean (SE): 9.4 (0.7) years	NR	53 (55)	Lanreotide microparticle formulation 30 mg: 97 (100) for a mean	NR	NR	NR	NR	NA (no patients had biochemical

Study	Treatment	N	Male, N (%)	Age, years, mean (SD)	Age, years, median (range)	Time since diagnosis	Previous surgery, N (%)	Previous RT, N (%)	Previous treatments, N (%)	GH level, µg/L, mean (SD)	GH level, µg/L, median (range)	IGF-I level, mean (SD)	IGF-I level, median (range)	Time with controlled IGF-I prior to EDI initiation
									(SE) of 3.1 (0.3) years					control at baseline)
Martinez-Delgado 2007 [21]	OCT 20 mg every 4 weeks	3	NR	NR	NR	NR	NR	NR	OCT 10–30 mg every 4 weeks: 3 (100) for ≥6 months	NR	NR	NR	NR	All ≥6 months
	OCT 20 mg every 8 weeks	6	NR	NR	NR	NR	NR	NR	OCT 10–30 mg every 4 weeks: 6 (100) for ≥6 months	NR	NR	NR	NR	All ≥6 months
	OCT 20 mg every 12 weeks	3	NR	NR	NR	NR	NR	NR	OCT 10–30 mg every 4 weeks: 3 (100) for ≥6 months	NR	NR	NR	NR	All ≥6 months
Ronchi 2007 [30]	LAN 120 mg every 8 weeks	6	NR	NR	NR	NR	NR	NR	OCT 10–30 mg every 4 weeks: 6 (100) for 6–18 months (inclusion criteria)	NR	NR	NR	NR	NA (no patients had biochemical control at baseline)
	LAN 120 mg every 6 weeks	4	NR	NR	NR	NR	NR	NR	OCT 10–30 mg every 4 weeks: 4 (100) for 6–18 months (inclusion criteria)	NR	NR	NR	NR	NA (no patients had biochemical control at baseline)
	LAN 120 mg every 4 weeks	12	NR	NR	NR	NR	NR	NR	OCT 10–30 mg every 4 weeks: 12 (100) for 6–18 months (inclusion criteria)	NR	NR	NR	NR	NA (no patients had biochemical control at baseline)
Schopohl 2011 [32]	LAN 120 mg every 4 weeks	17	NR (70.6)	50.9 (15)	NR	Mean (SD): 12.6 (9.6) years	NR	NR	OCT 30 mg: 17 (100) for a mean (SD) of 3.1 (2.6) years	NR	NR	NR	NR	All ≥6 months
	LAN 120 mg every 6 weeks	11	NR (18.2)	56.7 (11.8)	NR	Mean (SD): 10.2 (10.0) years	NR	NR	OCT 20 mg: 11 (100) for a mean (SD) of 2.9 (2.8) years	NR	NR	NR	NR	All ≥6 months
	LAN 120 mg every 8 weeks	7	NR (28.6)	53 (10.4)	NR	Mean (SD): 10.2 (5.2) years	NR	NR	OCT 10 mg: 7 (100) for a mean (SD) of 3.8 (2.6) years	NR	NR	NR	NR	All ≥6 months

Study	Treatment	N	Male, N (%)	Age, years, mean (SD)	Age, years, median (range)	Time since diagnosis	Previous surgery, N (%)	Previous RT, N (%)	Previous treatments, N (%)	GH level, µg/L, mean (SD)	GH level, µg/L, median (range)	IGF-I level, mean (SD)	IGF-I level, median (range)	Time with controlled IGF-I prior to EDI initiation
Turner 2004 [34]	OCT 20 mg every 12 weeks	3	NR	NR	NR (53–75)	NR	2 (NR)	0 (NR)	Subcutaneous octreotide, bromocriptine, or lanreotide acetate: NR	NR	NR (6.0–11.8) mU/L	NR	NR (29.6–56.5) nmol/L	NA (no patients had biochemical control at baseline)
	OCT 20 mg every 10 weeks	2	NR	NR	NR (69–81)	NR	0 (NR)	1 (NR)	Subcutaneous octreotide, bromocriptine, or lanreotide acetate: NR	NR	NR (7.7–8.2) mU/L	NR	NR (52.2–56.2) nmol/L	NA (no patients had biochemical control at baseline)
	OCT 20 mg every 8 weeks	6	NR	NR	NR (37–62)	NR	2 (NR)	3 (NR)	Subcutaneous octreotide, bromocriptine, or lanreotide acetate: NR	NR	NR (8.5–30.7) mU/L	NR	NR (35.3–83.4) nmol/L	NA (no patients had biochemical control at baseline)
	OCT 20–30 mg every 6 weeks	6	NR	NR	NR (47–72)	NR	2 (NR)	3 (NR)	Subcutaneous octreotide, bromocriptine, or lanreotide acetate: NR	NR	NR (8.0 – 26.0) mU/L	NR	NR (15.5–71.5) nmol/L	NA (no patients had biochemical control at baseline)
	OCT 20–30 mg every 4 weeks	2	NR	NR	NR (67–73)	NR	0 (NR)	1 (NR)	Subcutaneous octreotide, bromocriptine, or lanreotide acetate: NR	NR	NR (12.0–12.7) mU/L	NR	NR (24.5–67.7) nmol/L	NA (no patients had biochemical control at baseline)
Studies assessing pegvisomant														
Colao 2019 [8]	OCT 40 mg every 4 weeks	7	Female: 3 (42.9)	57.9 (7.7)	NR	Median (range): 32.0 (10–240) months	0 (0)	NR	LAN 120 mg every 4 weeks or OCT 30 mg every 4 weeks: 7 (100) for ≥6 months	NR	3.6 (2.6–14)	NR	477 (190–757) ng/mL	NA (no patients had biochemical control at baseline)
	OCT 40 mg every 4 weeks + pegvisomant 70 mg weekly	31	Female: 17 (54.8)	44.6 (10.5)	NR	Median (range): 47.0 (9–300) months	0 (0)	NR	LAN 120 mg every 4 weeks or OCT 30 mg every 4 weeks: 31 (100) for ≥6 months	NR	4.8 (3.0–77)	NR	643 (323–1069) ng/mL	NA (no patients had biochemical control at baseline)
	OCT 40 mg every 4 weeks + cabergoline 0.25–0.5mg twice	32	Female: 18 (56.3)	49.3 (10.5)	NR	Median (range):	0 (0)	NR	LAN 120 mg every 4 weeks or OCT 30 mg every 4 weeks: 32 (100) for ≥6 months	NR	5.7 (2.6–41)	NR	649 (310–1158) ng/mL	NA (no patients had biochemical

Study	Treatment	N	Male, N (%)	Age, years, mean (SD)	Age, years, median (range)	Time since diagnosis	Previous surgery, N (%)	Previous RT, N (%)	Previous treatments, N (%)	GH level, µg/L, mean (SD)	GH level, µg/L, median (range)	IGF-I level, mean (SD)	IGF-I level, median (range)	Time with controlled IGF-I prior to EDI initiation
	weekly, 4x weekly then daily (from Week 4)					36.5 (11–290) months								control at baseline)
Bonert 2020 [5]	Pegvisomant weekly 40–160 mg + LAN 120 mg or OCT 30 mg every 4 weeks<br>Pegvisomant weekly 40–160 mg + LAN 60 mg or OCT 10 mg every 4 weeks<br>Pegvisomant daily 15–60 mg + LAN 60 mg or OCT 10 mg every 4 weeks	52	20 (38)	49.5 (14.3)	NR	NR	35 (67)	NR	• LAN: 23 (44) for ≥3 months<br>• OCT: 22 (42) for ≥3 months<br>• Pegvisomant monotherapy, switched to SRL: 3 (6) for ≥3 months<br>• Combination SRL plus pegvisomant: 3 (6), 2 every week and 1 daily for ≥3 months<br>• Dopamine agonist monotherapy, switched to SRL: 1 (2) for ≥3 months	NR	NR	NR	NR	NR
PEGASO (Camara 2019) [6]	Pegvisomant non-daily (dosage and drug combination unclear)	108 (43 on EDI)	Female: 65 (60.2)	55.1 (14.5)	NR	Mean (SD): 11.3 (6.9) years	91 (85)	47 (43.9)	• SRL: 101 (94.4)<br>• Cabergoline: 44 (41.1)	NR	NR	NR	NR	NR
Higham 2009 [13]	Pegvisomant monotherapy 10–20 mg twice weekly<br>Pegvisomant monotherapy 10–20 mg weekly	7	4 (NR)	57 (7)	NR	Range: 3–16 months	6 (NR)	7 (NR)	Pegvisomant at a median (range) dose of 15 (10–20) mg once daily: 7 (100) for a median (range) of 24 (12–96) months	NR	NR	NR	NR	All ≥3 months
Franck 2015 [12]	Pegvisomant 80 mg weekly + OCT 30 mg or LAN 120 mg every 4 weeks	104	NR (58.7)	NR	NR	Median (IQR): 1.4 (0.9–3.5) years	NR (42.3)	NR (12.5)	• OCT 30 mg every 4 weeks: NR for ≥6 months<br>• LAN 120mg every 4 weeks: NR for ≥6 months	NR	4 (IQR: 2.3–10.3) µg/L	NR	1.81 (IQR:1.48–2.50) ×ULN	NA (no patients had biochemical control at baseline)
ACROSTUDY (Kuhn 2021) [16]	Pegvisomant monotherapy median dose	167	79 (47.3)	49.4 (13.4)	NR	NR	127 (76.0)	48 (28.7)	• SRL: 154 (92.2)<br>• Dopamine agonist: 67 (40.1)	NR	NR	NR	NR	NR

Study	Treatment	N	Male, N (%)	Age, years, mean (SD)	Age, years, median (range)	Time since diagnosis	Previous surgery, N (%)	Previous RT, N (%)	Previous treatments, N (%)	GH level, µg/L, mean (SD)	GH level, µg/L, median (range)	IGF-I level, mean (SD)	IGF-I level, median (range)	Time with controlled IGF-I prior to EDI initiation
	15 mg, 1–14 injections weekly													
	Pegvisomant primary bitherapy median dose 10 mg, 1–14 injections weekly	88	51 (58.0)	47.0 (16.0)	NR	NR	63 (71.6)	22 (25.0)	• SRL: 85 (96.6)<br>• Dopamine agonist: 39 (44.3)	NR	NR	NR	NR	NR
Madsen 2011 [20]	OCT 10–30 mg every 4 weeks or LAN 80 mg every 4 weeks	6	1 (NR)	52 (NR)	NR	NR	5 (83)	1 (17)	SRL: NR	NR	0.88 (0.18–2.3)	208.6 (18.5) µg/L	NR	NR
	Pegvisomant 15–30 mg twice weekly + OCT or LAN at half usual dosage	12	6 (NR)	55.2 (NR)	NR	NR	9 (75)	1(8)	SRL: NR	NR	0.72 (0.16–2.61)	221.0 (16.6) µg/L	NR	NR
Neggers 2009 [24]	Pegvisomant 20–200 mg weekly or twice weekly + OCT or LAN (dose not reported)	86	Female: 37 (NR)	54 (NR)	NR (19–83)	NR	44 (NR)	20	• OCT: 31 (NR) for ≥6 months<br>• LAN: 55 (NR) for ≥6 months	NR	NR	NR	NR	NR
Neggers 2008 [27]	Pegvisomant 40 mg weekly + LAN or OCT	20	Female: 9 (45)	55 (10)	56 (39–74)	NR	15 (75)	6 (30)	• LAN: 8 (40) for ≥36 months<br>• OCT: 12 (60) for ≥36 months	1.12 (0.7)	1 (0.2–3.1)	25.1 (5) nmol/L	24.6 (15.6–35.7) nmol/L	NR
Neggers 2007 [28]	Pegvisomant 60 mg once or twice weekly (40 mg starting dose) + LAN 120 mg or OCT 30 mg every 4 weeks	32	Female: 13 (41)	53 (12.8)	52 (30–81)	NR	• Transsphenoidal surgery and radiotherapy: 8 (25)<br>• Transsphenoidal surgery only: 6 (19)		• LAN 120mg every 4 weeks: 22 (69) for ≥6 months<br>• OCT 30 mg every 4 weeks: 10 (31) for ≥6 months	10.1 (14.2)	5.2 (0.4–69.8)	65 (28.9) nmol/L	60 (32–122) nmol/L	NA (no patients had biochemical control at baseline)
PAPE (Muhammad 2018) [22]	PAS-LAR monotherapy 60 mg every 4 weeks	15	NR	NR	NR	NR	NR	NR	NR	2.5 (NR)	NR	0.83 (NR) ×ULN	NR	NR
	PAS-LAR 60 mg every 4 weeks + pegvisomant	46	NR	NR	NR	NR	NR	NR	NR	11.5 (NR)	NR	1.01 (NR) ×ULN	NR	NA (no patients had biochemical

Study	Treatment	N	Male, N (%)	Age, years, mean (SD)	Age, years, median (range)	Time since diagnosis	Previous surgery, N (%)	Previous RT, N (%)	Previous treatments, N (%)	GH level, µg/L, mean (SD)	GH level, µg/L, median (range)	IGF-I level, mean (SD)	IGF-I level, median (range)	Time with controlled IGF-I prior to EDI initiation
	61 mg (starting dose) weekly													control at baseline)
	LAN 120 mg or OCT 30 mg + pegvisomant weekly	61	32 (NR)	NR	53 (26–80)	Median: 8.9 years	27 (44.2)	7 (11.5)	• LAN: 35 (57.4)<br>• OCT: 26 (42.6)<br>• Cabergoline: 2 (3.3)	9.3 (NR) µg/L	NR	0.97 (NR) ×ULN	NR	NR
Muhammad 2016 [23]	Pegvisomant monotherapy 60 mg (starting dose) once or twice weekly	15	NR	NR	58 (35–80)	NR	3 (20)	6 (40)	• LAN + weekly pegvisomant: 13 (87)<br>• OCT + weekly pegvisomant: 2 (13)<br>Median (range) weekly pegvisomant dose overall: 60 (9.3–33.4) mg	NR	3.03 (0.19–15.95)	NR	0.62 (0.3–0.84) ×ULN	All >6 months
van der Lely 2011 [35]	Pegvisomant 40–80 mg weekly to twice weekly + LAN 120 mg every 4 weeks	57	29 (50.9)	51.6 (12.7)	NR	Mean (SD): 7.7 (7.0) years	38 (66.7)	18 (31.6)	• LAN: 24 (42.1) for ≥6 months<br>• OCT: 20 (35.1) for ≥6 months<br>• Pegvisomant: 13 (22.8) for ≥3 months	NR	NR	3.36 (0.85) z-score	3.13 (2.04–6.03) z-score	NA (no patients had biochemical control at baseline)
Dassie 2019 [9]	Pegvisomant 24 mg daily	29[b]	NR	NR	NR	NR	NR	NR	NR	NR	NR	2.3 (NR) ×ULN	NR	NR
	Pegvisomant 12 mg non-daily	14	NR	NR	NR	NR	NR	NR	NR	NR	NR	1.95 (NR) ×ULN	NR	NR

Footnotes: [a]Calculated from reported data, 22% of 157, and rounded up from one decimal place; [b]calculated from reported data, 43–14. **Abbreviations:** EDI: extended-dosing interval; GH: growth hormone; IGF-I: insulin-like growth factor I; IQR: interquartile range; NA: not applicable; NR: not reported; LAN: lanreotide autogel/depot; OCT: octreotide long-acting release; PAS-LAR: pasireotide long-acting release; RT: radiotherapy; SD: standard deviation; SE: standard error; SRL: somatostatin receptor ligand; ULN: upper limit of normal.

References

1. Abrams P, Alexopoulou O, Abs R, Maiter D, Verhelst J (2007) Optimalization and cost management of lanreotide-autogel therapy in acromegaly. Eur J Endocrinol 157(5):571–577
2. Alvarez-Escola C, Venegas-Moreno EM, Garcia-Arnes JA, Blanco-Carrera C, Marazuela-Azpiroz M, Galvez-Moreno MA et al (2019) ACROSTART: A retrospective study of the time to achieve hormonal control with lanreotide autogel treatment in Spanish patients with acromegaly. [ACROSTART: Estudio retrospectivo del periodo de tiempo para lograr el control hormonal con lanreotida Autogel en pacientes con acromegalia en la practica clinica espanola.]. Endocrinol Diabetes Nutr 66(5):320–329
3. Bernabéu I, Fajardo C, Marazuela M, Cordido F, Venegas EM, de Pablos-Velasco P et al (2020) Effectiveness of lanreotide autogel 120 mg at extended dosing intervals for acromegaly. Endocrine 70(3):575–583
4. Biermasz NR, van den Oever NC, Frolich M, Arias AM, Smit JW, Romijn JA et al (2003) Sandostatin LAR in acromegaly: a 6-week injection interval suppresses GH secretion as effectively as a 4-week interval. Clin Endocrinol 58(3):288–295
5. Bonert V, Mirocha J, Carmichael J, Yuen KCJ, Araki T, Melmed S (2020) Cost-effectiveness and efficacy of a novel combination regimen in acromegaly: a prospective, randomized trial. J Clin Endocrinol Metab 105(9):E3236–E3245
6. Camara R, Venegas E, Garcia-Arnes JA, Cordido F, Aller J, Samaniego ML et al (2019) Treatment adherence to pegvisomant in patients with acromegaly in Spain: PEGASO study. Pituitary 22(2):137–145
7. Colao A, Auriemma RS, Rebora A, Galdiero M, Resmini E, Minuto F et al (2009) Significant tumour shrinkage after 12 months of lanreotide autogel-120 mg treatment given first-line in acromegaly. Clin Endocrinol 71(2):237–245
8. Colao A, Zgliczyński W, Komorowski J, Kos-Kudła B, Tabarin A, Kerlan V et al (2019) Efficacy and safety of high-dose long-acting repeatable octreotide as monotherapy or in combination with pegvisomant or cabergoline in patients with acromegaly not adequately controlled by conventional regimens: results of an open-label, multicentre study. Endokrynol Pol 70(4):305–312
9. Dassie F, Rosson M, Parolin M, Russo L, Mazzocut S, Martini C et al (2019) Pegvisomant: daily versus non daily administration a single centre real life study. Endocrine Abstracts 63P1101
10. Espinosa-de-los-Monteros AL, Gonzalez B, Vargas G, Sosa E, Mercado M (2015) Octreotide LAR treatment of acromegaly in "real life": long-term outcome at a tertiary care center. Pituitary 18(3):290–296
11. Franck SE, Korevaar TIM, Petrossians P, Daly AF, Chanson P, Jaffrain-Réa ML et al (2017) A multivariable prediction model for pegvisomant dosing: monotherapy and in combination with long-acting somatostatin analogues. Eur J Endocrinol 176(4):421–431
12. Franck SE, van der Lely AJ, Delhanty PJ, Jorgensen JO, Neggers SJ (2015) Pegvisomant in combination with long-acting somatostatin analogues in acromegaly: the role of the GH receptor deletion of exon 3. Eur J Endocrinol 173(5):553–561
13. Higham CE, Thomas JD, Bidlingmaier M, Drake WM, Trainer PJ (2009) Successful use of weekly pegvisomant administration in patients with acromegaly. Eur J Endocrinol 161(1):21–25
14. Jehle S, Reyes CM, Sundeen RE, Freda PU (2005) Alternate-day administration of pegvisomant maintains normal serum insulin-like growth factor-I levels in patients with acromegaly. J Clin Endocrinol Metab 90(3):1588–1593
15. Khairi S, Sagvand BT, Pulaski-Liebert KJ, Tritos NA, Klibanski A, Nachtigall LB (2017) Clinical outcomes and self-reported symptoms in patients with acromegaly: an 8-year follow-up of a lanreotide study. Endocr Pract 23(1):56–65

16. Kuhn E, Caron P, Delemer B, Raingeard I, Lefebvre H, Raverot G et al (2021) Pegvisomant in combination or pegvisomant alone after failure of somatostatin analogs in acromegaly patients: an observational French ACROSTUDY cohort study. Endocrine 71(1):158–167
17. Lasolle H, Ferriere A, Vasiljevic A, Eimer S, Nunes ML, Tabarin A (2019) Pasireotide-LAR in acromegaly patients treated with a combination therapy: a real-life study. Endocr Connect 8(10):1383–1394
18. Lombardi G, Minuto F, Tamburrano G, Ambrosio MR, Arnaldi G, Arosio M et al (2009) Efficacy of the new long-acting formulation of lanreotide (Lanreotide autogel) in somatostatin analogue-naive patients with acromegaly. J Endocrinol Invest 32(3):202–209
19. Lucas T, Astorga R, Almeida R, Paiva, Ribeiro, Lisbona et al (2006) Efficacy of lanreotide autogel administered every 4-8 weeks in patients with acromegaly previously responsive to lanreotide microparticles 30 mg: A phase III trial. Clin Endocrinol 65(3):320–326
20. Madsen M, Poulsen PL, Orskov H, Møller N, Jørgensen JO (2011) Cotreatment with pegvisomant and a somatostatin analog (SA) in SA-responsive acromegalic patients. J Clin Endocrinol Metab 96(8):2405–2413
21. Martinez-Delgado IA, Gomez-Martinez G (2007) Usefulness of GH and IGF-1 to establish the dose and frequency of application of octreotide to treat acromegaly. [Spanish]. [IGF-1 y hormona de crecimiento. Marcadores para la aplicacion de octreotida en acromegalia.]. Rev Med Inst Mex Seguro Soc 45(3):291–295
22. Muhammad A, van der Lely AJ, Delhanty PJD, Dallenga AHG, Haitsma IK, Janssen J et al (2018) Efficacy and safety of switching to pasireotide in patients with acromegaly controlled with pegvisomant and first-generation somatostatin analogues (PAPE study). J Clin Endocrinol Metab 103(2):586–595
23. Muhammad A, Van Der Lely AJ, O'Connor R D, Delhanty PJ, Dal J, Dallenga AH et al (2016) What is the efficacy of switching to weekly pegvisomant in acromegaly patients well controlled on combination therapy? Eur J Endocrinol 174(5):663–667
24. Neggers SJ, de Herder WW, Janssen JA, Feelders RA, van der Lely AJ (2009) Combined treatment for acromegaly with long-acting somatostatin analogs and pegvisomant: long-term safety for up to 4.5 years (median 2.2 years) of follow-up in 86 patients. Eur J Endocrinol 160(4):529–533
25. Neggers SJ, Franck SE, de Rooij FW, Dallenga AH, Poublon RM, Feelders RA et al (2014) Long-term efficacy and safety of pegvisomant in combination with long-acting somatostatin analogs in acromegaly. J Clin Endocrinol Metab 99(10):3644–3652
26. Neggers SJ, Pronin V, Balcere I, Lee MK, Rozhinskaya L, Bronstein MD et al (2015) Lanreotide autogel 120 mg at extended dosing intervals in patients with acromegaly biochemically controlled with octreotide LAR: the LEAD study. Eur J Endocrinol 173(3):313–323
27. Neggers SJ, van Aken MO, de Herder WW, Feelders RA, Janssen JA, Badia X et al (2008) Quality of life in acromegalic patients during long-term somatostatin analog treatment with and without pegvisomant. J Clin Endocrinol Metab 93(10):3853–3859
28. Neggers SJ, van Aken MO, Janssen JA, Feelders RA, de Herder WW, van der Lely AJ (2007) Long-term efficacy and safety of combined treatment of somatostatin analogs and pegvisomant in acromegaly. J Clin Endocrinol Metab 92(12):4598–45601
29. Ramírez C, Vargas G, González B, Grossman A, Rábago J, Sosa E et al (2012) Discontinuation of octreotide LAR after long term, successful treatment of patients with acromegaly: is it worth trying? Eur J Endocrinol 166(1):21–26
30. Ronchi CL, Boschetti M, Uberti ECD, Mariotti S, Grottoli S, Loli P et al (2007) Efficacy of a slow-release formulation of lanreotide (autogel 120 mg) in patients with acromegaly previously treated with octreotide long acting release (LAR): An open, multicentre longitudinal study. Clin Endocrinol 67(4):512–519
31. Sagvand BT, Khairi S, Haghshenas A, Swearingen B, Tritos NA, Miller KK et al (2016) Monotherapy with lanreotide depot for acromegaly: long-term clinical experience in a pituitary center. Pituitary 19(4):437–447

32. Schopohl J, Strasburger CJ, Caird D, Badenhoop K, Beuschlein F, Droste M et al (2011) Efficacy and acceptability of lanreotide autogel 120 mg at different dose intervals in patients with acromegaly previously treated with octreotide LAR. Exp Clin Endocrinol Diabetes 119(3):156–162
33. Sesmilo G, Resmini E, Bernabeu I, Aller J, Soto A, Mora M et al (2014) Escape and lipodystrophy in acromegaly during pegvisomant therapy, a retrospective multicentre Spanish study. Clin Endocrinol 81(6):883–890
34. Turner HE, Thornton-Jones VA, Wass JA (2004) Systematic dose-extension of octreotide LAR: the importance of individual tailoring of treatment in patients with acromegaly. Clin Endocrinol 61(2):224–231
35. Van Der Lely AJ, Bernabeu I, Cap J, Caron P, Colao A, Marek J et al (2011) Coadministration of lanreotide autogel and pegvisomant normalizes IGF1 levels and is well tolerated in patients with acromegaly partially controlled by somatostatin analogs alone. Eur J Endocrinol 164(3):325–333
36. Vilar L, Fleseriu M, Naves LA, Albuquerque JL, Gadelha PS, dos Santos Faria M et al (2014) Can we predict long-term remission after somatostatin analog withdrawal in patients with acromegaly? Results from a multicenter prospective trial. Endocrine 46(3):577–584
